# PIGL promotes the docetaxel resistance of prostate cancer and is regulated by E3 ubiquitin ligase HUWE1

**DOI:** 10.3389/fimmu.2026.1740132

**Published:** 2026-06-18

**Authors:** Pan Gao, Bo-Han Lin, Zhi Zhang, Ze-Yu Yi, Zhi-Bin Ke, Zhong-Hua Zhu, Zhen Kang, Yong Wei

**Affiliations:** 1 Department of Urology, Urology Research Institute, The First Affiliated Hospital, Fujian Medical University, Fuzhou, China; 2 Department of Urology, National Regional Medical Center, Binhai Campus of the First Affiliated Hospital, Fujian Medical University, Fuzhou, China; 3 Department of Urology, Second People’s Hospital of Yichang, Yichang, China; 4 Department of Urology, Second People’s Hospital of China Three Gorges University, Yichang, China; 5 Department of Urology, The First People’s Hospital of Yunnan Province, Kunming, China; 6 Department of Urology, The Affiliated Hospital of Kunming University of Science and Technology, Kunming, China

**Keywords:** docetaxel resistance, HUWE1 gene, PIGL gene, prostate cancer, ubiquitination

## Abstract

**Introduction:**

The mechanism related to docetaxel resistance in PCa has not been fully elucidated, and in-depth study of the specific mechanism of docetaxel resistance in PCa is of great clinical significance for the treatment of PCa.

**Methods:**

We explored the expression level of PIGL and HUWE1 using TCGA database and our clinical sample. CCK8 assay, Transwell assay, colony formation assay, wound-healing assay were used to explore the role of HUWE1/PIGL. Ubiquitination assay and Co-immunoprecipitation assay were used to investigate the molecular mechanism.

**Results:**

We found that both the mRNA and protein of PIGL is highly expressed in prostate cancer (PCa) and associated with poor prognosis. The protein rather than mRNA of PIGL is highly expressed in docetaxel-resistant PCa and associated with prognosis in patients receiving docetaxel-based chemotherapy. High expression level of PIGL promotes the proliferation, migration, invasion, and docetaxel resistance of PCa cells *in vitro*. Mass spectrometry after immunoprecipitation identified HUWE1 as a binding protein of PIGL. The protein of HUWE1 is lowly expressed in docetaxel-resistant PCa and associated with prognosis in patients receiving docetaxel-based chemotherapy. HUWE1 might be served as the E3 ubiquitin ligase of PIGL and negatively regulated the expression of PIGL protein rather than mRNA. The knockdown of HUWE1 could promote the docetaxel resistance of PCa and could be rescued by PIGL silencing.

**Discussion:**

These findings emphasized the vital role of PIGL in the progression and docetaxel resistance of PCa and the effect of the E3 ubiquitin ligase HUWE1 on regulating the protein expression of PIGL.

## Introduction

Prostate cancer (PCa) is the most common malignant tumor in male genitourinary system, and its incidence has increased significantly in some coastal areas of China in recent years (1, 2). Localized PCa has good therapeutic effect, but many PCa patients in our country have developed to advanced stage when diagnosed, and advanced PCa is a difficult point in clinical treatment (3, 4). Endocrine therapy is the basic treatment of metastatic hormone-sensitive PCa (mHSPC); however, castration resistant PCa (CRPC) will eventually develop after a period of endocrine treatment (5, 6). The treatment of CRPC is a thorny issue for clinicians.

Docetaxel has been proved to play a role in preventing de-polymerization of microtubules through binding to β subunits of tubulins (7). Treatment with docetaxel leads to the apoptosis and mitotic arrest of cancer cells (8). Systemic chemotherapy based on docetaxel is an important neoadjuvant therapy for locally advanced PCa and an effective treatment for advanced PCa (including mHSPC and CRPC), but some PCa patients are prone to drug resistance to docetaxel, and chemotherapy resistance is a thorny problem in clinical treatment of advanced PCa (9, 10). At present, the mechanism related to docetaxel resistance in PCa has not been fully elucidated, and in-depth study of the specific mechanism of docetaxel resistance in PCa is of great clinical significance for the treatment of PCa (7).

PIGL encodes an enzyme that catalyzes the second step of glycosylphosphatidylinositol (GPI) biosynthesis, which is the de-N-acetylation of N-acetylglucosaminyl-phosphatidylinositol (GlcNAc-PI) (11, 12). It was reported that PIGL plays a role in disrupting the interaction between cMyc/BRD4 on the distant promoter of target genes and thus decreased the expression of CCL2 and CCL20 to reprogram the tumor microenvironment (TME) (11). Besides, the nuclear PIGL intensity or the change in PIGL-Y81 phosphorylation could be served as a biomarker to guide lenvatinib with PD-1 blockade therapy of hepatocellular carcinoma (11). As a key enzyme in GPI biosynthesis, the absence or dysfunction of PIGL would prevent GPI-anchored proteins from correctly anchoring onto the cell membrane. These proteins include many receptors, adhesion molecules and enzymes, which may be involved in drug endocytosis, exocytosis or signal transduction. However, there were no study exploring the role of PIGL in the docetaxel resistance of prostate cancer. In this study, we explored the vital role of PIGL in development and docetaxel resistance of PCa and identified HUWE1 as a E3 ubiquitin ligase of PIGL. These findings emphasized the vital role of PIGL and HUWE1 in the progression and docetaxel resistance of PCa and would contribute to the potential mechanism of resistance to docetaxel chemotherapy in PCa patients.

## Material and methods

### Cell culture

We purchased normal human epithelial cells (RWPE-1), human prostate cancer cell lines including DU145, PC-3, 22RV1, LnCap, C4-2, C4-2B from Procell Life Science & Technology Co., Ltd (Wuhan, China). RWPE-1, 22RV1, C4-2, C4-2B were cultured with RPMI-1640 medium added with 10% fetal bovine serum (FBS). DU145 was cultured with MEM medium added with 10% FBS. PC-3 was cultured with F-12K medium with 10% FBS. All cells were incubated in 5% CO_2_ at 37 °C.

### Clinical specimens

Fresh postoperative PCa tissues and/or paired tissues adjacent to PCa in the First Affiliated Hospital of Fujian Medical University were obtained for RNA extraction or protein extraction. Biopsy tissues of mCRPC patients receiving docetaxel chemotherapy in the First Affiliated Hospital of Fujian Medical University and the Second People’s Hospital of Yichang were obtained for RNA and protein extraction. We divided all enrolled patients into longer PFS and shorter PFS based on the median value of PFS.

### The cancer genome atlas-PRAD dataset

We analyzed the TCGA-PRAD dataset using the UALCAN online tool, which enables survival analysis and expression levels analysis in tumor subgroups. Level 3 RNA-seq and clinical data were used in the analyses.

### RT-qPCR analysis

We utilized TRIzol reagent to extract the total RNA and used the HiScript III All-in-one RT SuperMix Perfect (Vazyme, Cat: R333-01) to perform reverse transcription and generate cDNA. The reverse transcription reaction conditions were 50°C for 15min and then 85°C for 5 sec according to manufacturer’s specifications. The amount of total RNA used for cDNA synthesis was 1 μg. Next, we used Taq Pro Universal SYBR qPCR Master Mix (Vazyme, Code: Q712-03) to detect the expression levels using QuantStudio™ 5 real-time PCR system (Applied Biosystems). According to manufacturer’s specifications, the reaction conditions were 1 cycle of 95 °C for 30 sec (polymerase activation), and then 40 cycles of 95 °C for 10 sec (denaturation) and 40 cycles of 60 °C for 30 sec (annealing/extension). The expression levels were normalized based on the expression of GAPDH.

### Western blot

The RIPA buffer was used to lyse cell or tissue samples, and the BCA assay kit (P0010, Beyotime) was used to quantify proteins. The loading amount of total protein per lane was 50 μg. Proteins were separated in 10% SDS-PAGE gels and transferred to PVDF membranes. Then, the membranes were incubated with primary antibodies overnight at 4°C, which was followed by incubation with HRP-conjugated goat anti-rabbit or anti-mouse IgG for 1 hour. The protein bands were exposed using enhanced chemiluminescence reagent. Primary antibodies used were: anti-PIGL (1:1000, Rabbit, Polyclonal, Immunoway, YT6358), anti-HUWE1 (1:1000, Rabbit, Polyclonal, Proteintech, 19430-1-AP), anti-polyubiquitin (1:1000, Rabbit, Polyclonal, CST, 58395), anti-β-actin (1:2000, Rabbit, Polyclonal, Proteintech, 20536-1-AP), anti-GAPDH (1:2000, Mouse, Monoclonal, Proteintech, 60004-1-Ig).

### Lentivirus transduction

We purchased Lentivirus carrying shRNA targeting PIGL or HUWE1, or plasmids of PIGL or HUWE1 from Shanghai GENECHEM Co., Ltd (Shanghai, China). The lentivirus was used to infect PC-3 and DU145 cells accompanied with Polybrene. We then cultured cells in medium-containing puromycin for the selection of stable clones.

Sequence for shNC: 5’-CTCGCTTGGGCGAGAGTAA-3’.
Sequence for shPIGL: #1, 5’-AATCATTACACTGGAGAGT-3’;
#2, 5’-TTAGGTAACTTCCCTTCTG-3’.
Sequence for shHUWE1: #1, 5’-CGACGAGAACTAGCACAGAAT-3’;
#2, 5’-CCAGCTTCTAACATCCGTGTT-3’.

### Construction of PC-3R and DU145R

To establish docetaxel-resistant prostate cancer cell lines, PC-3 and DU145 cells were subjected to an intermittent, stepwise dose-escalation protocol. Briefly, when cells reached approximately 90% confluence, they were exposed to 0.1 nM DTX for 24 hours. Following induction, the medium was replaced with drug-free complete medium, and culture continued until normal proliferative activity resumed. Cells were then re-challenged with the same DTX concentration to confirm adaptation. The docetaxel concentration was subsequently increased incrementally through the following series: 0.2, 0.5, 0.75, 1, 1.5, 2, 5, 7.5, 10, 15, 20, and 40 nM. This cycle of induction, recovery, and re-challenge was repeated at each new concentration. After approximately six months of continuous selection, stably resistant populations emerged, designated as PC-3R and DU145R. The acquired DTX resistance was functionally validated by determining the half-maximal inhibitory concentration (IC50) of DTX and by assessing colony-forming capacity in the presence of DTX.

### Colony formation assay

We plated cells into six-well plates with a density of 1000 cells per well. Next, the cells were incubated for 2 weeks under docetaxel treatment, which was followed by fixing with 4% formaldehyde and staining with 0.5% crystal violet. Last, we calculated the number of colonies.

### Cell counting kit-8 assay

For CCK8 proliferation assay, stable DU145 cells and PC-3 cells were seeded in 96-well plate with 5000 cells per 100 μl (five duplicate wells/each group). After 0, 24, 48, 72, 96 hours of cell culture, mediums were exchanged for fresh mediums containing 10% CCK-8 reagent. Then, the 96-well plate was placed at 37 °C for 1 hour. The absorbances were determined at 450 nm using a SpectraMax M5 microplate reader (Molecular Devices, Sunnyvale, CA, USA).

For CCK8 toxicity assays, stable DU145 cells and PC-3 cells were seeded in 96-well plate with 10000 cells per 100 μl (five duplicate wells/each group). After 24 hours, cells were incubated with docetaxel (concentration gradients: 2.5nM, 5nM, 7.5nM, 10nM, 12.5nM, 15nM, 17.5nM, 20nM) for 24 hours. Next, mediums were exchanged for fresh mediums containing 10% CCK-8 reagent and the plates were incubated for 1 hour at 37 °C. The absorbances were determined at 450 nm using a SpectraMax M5 microplate reader (Molecular Devices, Sunnyvale, CA, USA).

### Transwell migration and invasion assay

Stable DU145 cells and PC-3 cells were starved for 48 hours with serum-free medium. We planted cells into the upper well at 4×10 (4) cells and added medium containing 30% FBS was into the lower well. For invasion assay, the Matrigel Matrix (Corning) were coated in the upper well before planting cells. Cells were allowed to migrate or invade for 24 to 48 hours. We use 4% paraformaldehyde and 1% crystal violet for cell fixation and staining separately. Three random microscopic fields were selected and photographed.

### Wound-healing assay

Stable DU145 cells and PC-3 cells were seeded into 6-well plate at 3×10 (5) cells per well. when achieved 90% confluence, we used a 1 ml gun head to scratched vertically the cells. The cells images were captured at 0 hour and 24 hours. The cell migration at the same location was recoded at 24 hours with inverted microscope.

### Ubiquitination assay

Cells, following the transfection with HUWE1-targeting shRNAs or shNC, were incubated with 10 µM MG132 (a proteasome inhibitor) for 6 hours prior to harvest to stabilize ubiquitinated proteins. Total cellular proteins were then extracted using RIPA lysis buffer supplemented with a cocktail of protease inhibitors. For immunoprecipitation, equal amounts of protein lysates were pre-cleared and subsequently incubated with a specific anti-PIGL antibody (PIGL, immunoway, YT6358) overnight at 4 °C, followed by the addition of Protein A/G magnetic beads for 2 hours. The immunocomplexes were extensively washed with ice-cold lysis buffer to remove non-specifically bound proteins. The resulting eluates, along with corresponding whole-cell lysate inputs, were resolved by SDS-PAGE and transferred onto PVDF membranes. The ubiquitination level of PIGL was then detected by western blotting using an anti-ubiquitin antibody.

### Co-immunoprecipitation assay

Cells were lysed in lysis buffer containing protease and phosphatase inhibitors. Protein concentrations were quantified, and equal amounts of lysate were incubated with either primary antibody (HUWE1, proteintech, 19430-1-AP) or control IgG overnight at 4 °C. Immunocomplexes were captured using Protein A/G agarose beads for 2 to 4 h at 4 °C. After extensive washing, bound proteins were eluted by boiling in SDS loading buffer and analyzed by western blotting.

### Silver staining and mass spectrometry

The IP lysis buffer containing protease inhibitor was added into the indicated cells. The cell lysate was incubated with a specific antibody or IgG. Next, protein A/G magnetic beads were incubated with the lysate and the beads coupled to the antigen-antibody complexes were washed with IP lysis buffer. The product was subjected to liquid chromatography mass spectrometry/mass spectrometry (LC–MS/MS). The silver staining was performed using the Fast Silver Stain Kit (Beyotime, Haimen, China) according to the standard protocol.

### Statistical analysis

All data represents at least 3 independent experiments and are shown as mean ± SD. Statistical analysis was performed with GraphPad Prism 8.0. If not mentioned otherwise in the figure legends, statistical significance (*P < 0.05; **P < 0.01; ***P < 0.001) was determined by unpaired or paired two-tailed Student’s t tests, two-way repeated measures ANOVA test, one-way ANOVA test, or Wilcoxon rank sum test where appropriate. Repeated measures ANOVA was conducted to assess repeated measurement data. Survival analysis was conducted by Gehan-Breslow-Wilcoxon test.

## Results

### PIGL is highly expressed in PCa and associated with poor prognosis

**Figure 1:**
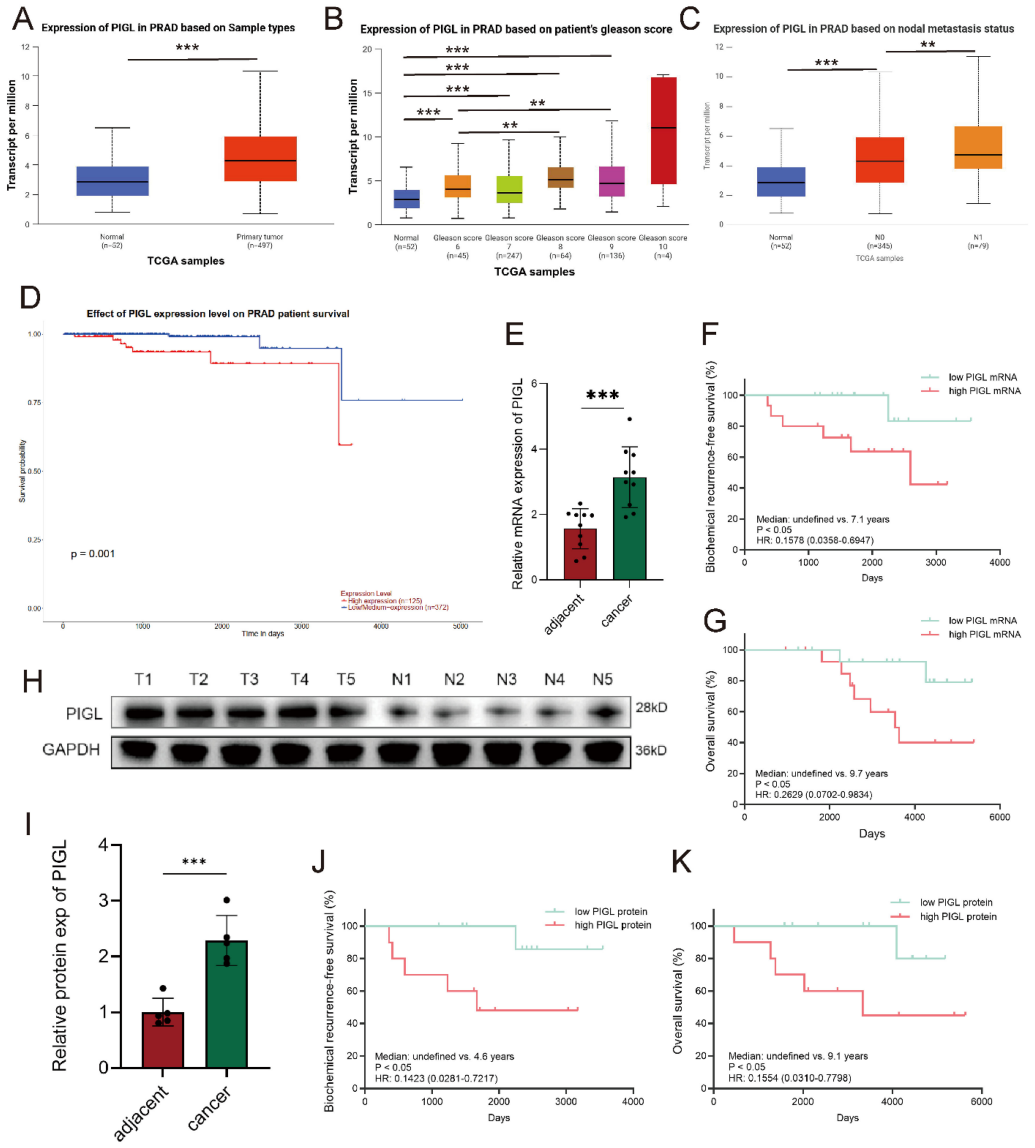
PIGL is highly expressed in PCa and associated with poor prognosis. **(A)** The differential expression of PIGL mRNA between normal and PCa tissues analyzed by TCGA-PARD dataset. **(B)** The association of expression level of PIGL mRNA with Gleason score analyzed by TCGA-PARD dataset. **(C)** The association of expression level of PIGL mRNA with nodal metastasis analyzed by TCGA-PARD dataset. **(D)** The association of expression level of PIGL mRNA with overall survival (OS) analyzed by TCGA PARD dataset. **(E)** RT-qPCR showing the differential expression of PIGL mRNA between PCa tissues and paired tissues adjacent to cancer from patients in our centers; paired two-tailed student’s t-test. **(F, G)** The association of expression level of PIGL mRNA in PCa tissues from patients in our centers with biochemical recurrence-free survival (BCRFS) and OS; Kaplan-Meier analysis Gehan-Breslow-Wilcoxon test. **(H–I)** Western blot and the quantitative results showing the differential expression of PIGL protein between PCa tissues and paired tissues adjacent to cancer from patients in our centers; paired two-tailed student’s t-test. **(J, K)** The association of expression level of PIGL protein in PCa tissues from patients in our centers with BCRFS and OS; Kaplan-Meier analysis followed by Gehan-Breslow-Wilcoxon test. **P < 0.01, ***P < 0.001.

The role of PIGL in the development and docetaxel resistance of prostate cancer remains uncertain. In this study, we analyzed the data from TCGA-PARD dataset and found that the mRNA expression level of PIGL is highly increased in PCa tissues compared with normal tissues (**Figure 1A**). Moreover, there was a positive association of PIGL mRNA expression level with Gleason score in TCGA-PARD dataset (**Figure 1B**) and the mRNA expression level of PIGL is highly increased in PCa tissues of nodal metastasis compared with PCa tissues of non-nodal metastasis (**Figure 1C**). The data from TCGA-PARD dataset revealed that high expression level of PIGL mRNA was associated with poor overall survival (**Figure 1D**). We conducted RT-qPCR to detect the mRNA expression level of PIGL in 10 cases of PCa tissues and paired tissues adjacent to cancer in our centers, which revealed that the mRNA expression level of PIGL is highly increased in PCa tissues compared with paired tissues adjacent to cancer (**Figure 1E**). The data from our center revealed that high expression level of PIGL mRNA was associated with poor biochemical recurrence-free survival and overall survival (**Figures 1F, G**). We conducted western blot to detect the protein expression level of PIGL in 5 cases of PCa tissues and paired tissues adjacent to cancer in our centers, which revealed that the protein expression level of PIGL is highly increased in PCa tissues compared with paired tissues adjacent to cancer (**Figures 1H, I**). The data from our center revealed that high expression level of PIGL protein was associated with poor biochemical recurrence-free survival and overall survival (**Figures 1J, K**). These results demonstrated that the mRNA and protein of PIGL is highly expressed in PCa and associated with poor prognosis.

### Construction of stable PCa cell lines with PIGL overexpression or knockdown

To further reveal the role of PIGL in the development of PCa and the docetaxel resistance, we constructed the stable PCa cell lines with PIGL overexpression or knockdown. Firstly, we conducted the RT-qPCR and western blot to explore the mRNA and protein expression level of PIGL in different PCa cell lines (**Supplementary Figures 1A, B**). The results showed that the mRNA and protein expression level of PIGL was highest in PC-3 and lowest in DU145; hence, we overexpressed PIGL in DU145 and knockdown PIGL in PC-3 to construct stable DU145 cell lines with PIGL overexpression or stable PC-3 cell lines with knockdown. According to the results of RT-qPCR, we selected the two interfered targets with the highest interference efficiencies in PC-3. Western blot analysis confirmed that the expression level of PIGL protein were knockdown by using corresponding shRNAs in PC-3. According to the results of RT-qPCR and western blot, we successfully constructed the stable DU145 cell lines with PIGL overexpression (**Supplementary Figures 1C, D**). These results showed that we successfully constructed the stable PC-3 cell lines with PIGL knockdown and the stable DU145 cell lines with PIGL overexpression.

### High expression of PIGL promotes the proliferation, migration and invasion ability of PCa cells *in vitro*

**Figure 2:**
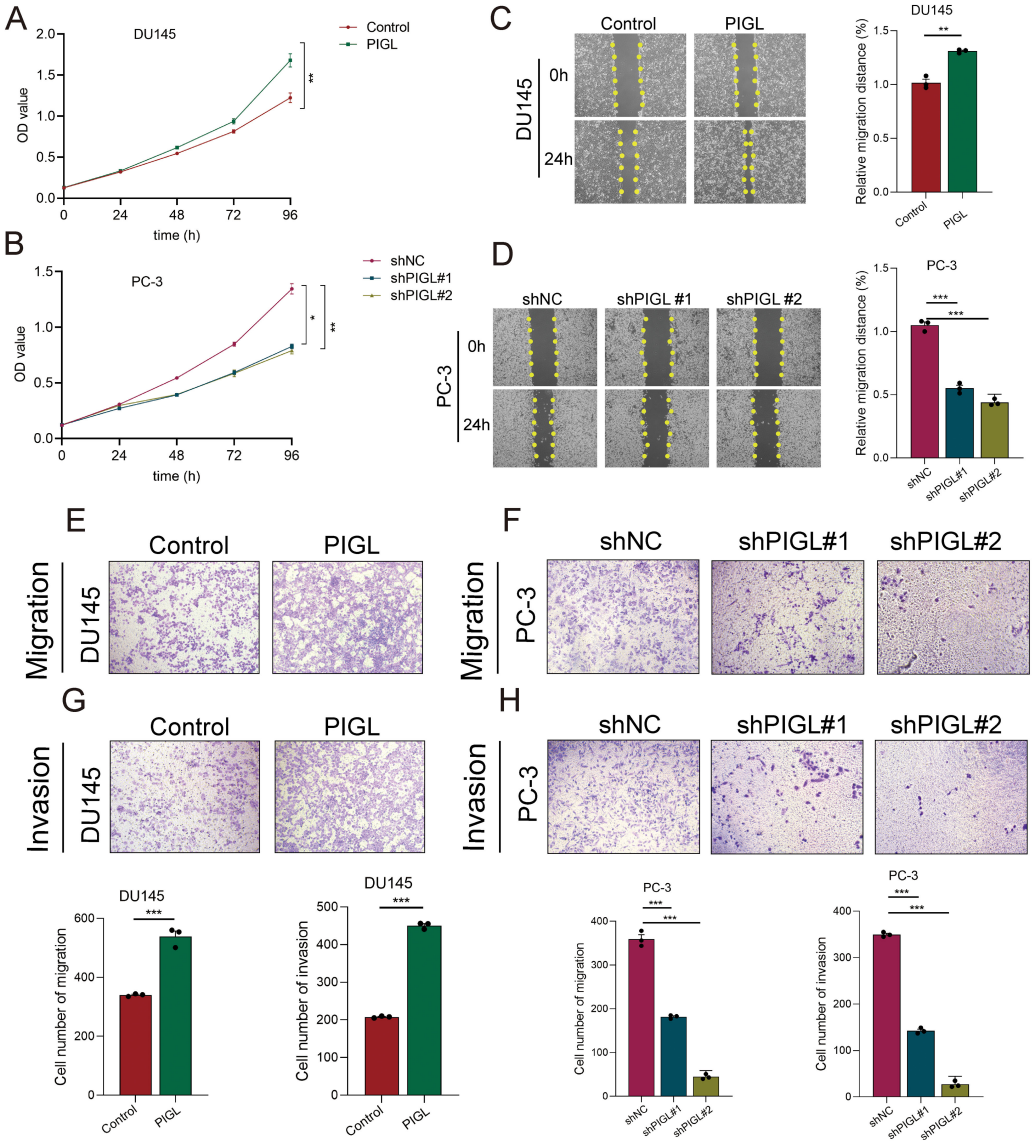
High expression of PIGL promotes the proliferation, migration, and invasion of PCa cells *in vitro.* **(A)** CCK8 proliferation assay indicating the effect of PIGL overexpression on the cell viability of DU145 cells; two-way repeated measures ANOVA test. **(B)** CCK8 proliferation assay indicating the effect of PIGL knockdown on the cell viability of PC-3 cells; two-way repeated measures ANOVA test. **(C)** Representative images and quantitative results of wound healing assay showing the effect of PIGL overexpression on the migration ability of DU145 cells; unpaired two-tailed student’s t-test. **(D)** Representative images and quantitative results of wound healing assay showing the effect of PIGL knockdown on the migration ability of PC-3 cells; one-way ANOVA test. **(E)** Representative images and quantitative results of Transwell migration assay showing the effect of PIGL overexpression on the migration ability of DU145 cells; unpaired two-tailed student’s t-test. **(F)** Representative images and quantitative results of Transwell migration assay showing the effect of PIGL knockdown on the migration ability of PC-3 cells; one-way ANOVA test. **(G)** Representative images and quantitative results of Transwell invasion assay showing the effect of PIGL overexpression on the invasion ability of DU145 cells; unpaired two-tailed student’s t-test. **(H)** Representative images and quantitative results of Transwell invasion assay showing the effect of PIGL knockdown on the invasion ability of PC-3 cells; one-way ANOVA test. *P < 0.05, **P < 0.01, ***P < 0.001.

The results of CCK8 proliferation assay indicated that the overexpression of PIGL promotes the cell viability of DU145 cells and the knockdown of PIGL inhibits the cell viability of PC-3 cells (**Figures 2A, B**). The results of wound healing assay indicated that the overexpression of PIGL promotes the migration ability of DU145 cells and the knockdown of PIGL inhibits the migration ability of PC-3 cells (**Figures 2C, D**). The results of Transwell migration assay showed that the overexpression of PIGL promotes the migration ability of DU145 cells and the knockdown of PIGL inhibits the migration ability of PC-3 cells (**Figures 2E, F**). The results of Transwell invasion assay showed that the overexpression of PIGL promotes the invasion ability of DU145 cells and the knockdown of PIGL inhibits the invasion ability of PC-3 cells (**Figures 2G, H**). Besides, PIGL overexpression would increase the CCL2 and CCL20 mRNA expression levels in DU145 cells, CCL2 and CCL20 content in the culture medium of DU145 cells, while PIGL knockdown would decrease, the CCL2 and CCL20 mRNA expression levels in PC-3 cells, the CCL2 and CCL20 content in the culture medium of PC-3 cells (**Supplementary Figure 2**). These results showed that high expression of PIGL promotes the proliferation, migration and invasion ability of PCa cells and low expression of PIGL inhibits the proliferation, migration and invasion ability of PCa cells.

### The expression level of PIGL protein rather than mRNA predicts the docetaxel resistance of PCa

**Figure 3:**
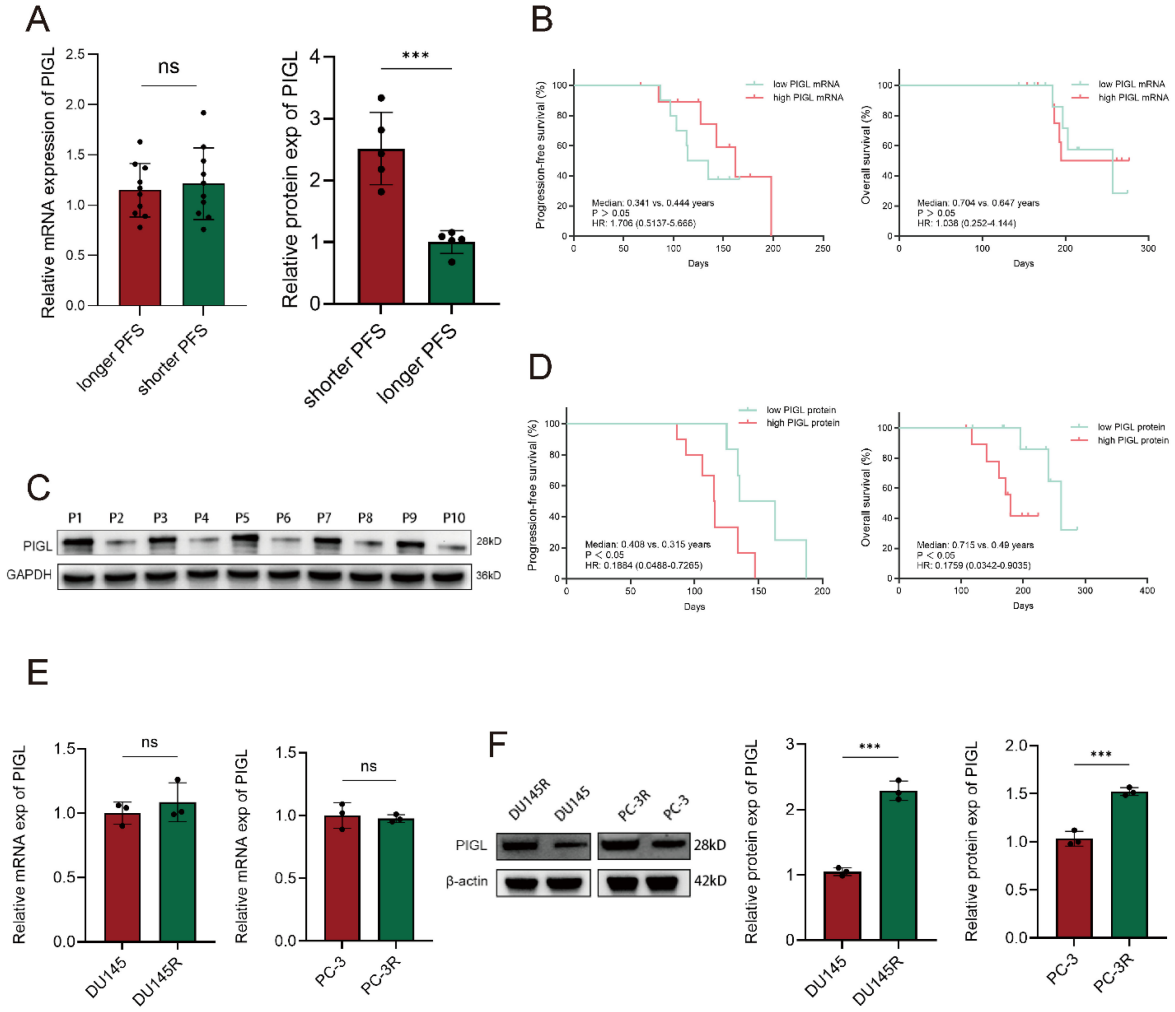
The expression level of PIGL protein rather than mRNA was increased in the docetaxel-resistant PCa cells and tissues. **(A)** RT-qPCR showing the differential expression of PIGL mRNA between longer progression-free survival (PFS) and shorter PFS PCa biopsy tissues from mCRPC patients receiving docetaxel chemotherapy in our centers; unpaired two-tailed student’s t-test. **(B)** The association of expression level of PIGL mRNA with PFS and overall survival (OS) in PCa biopsy tissues from mCRPC patients receiving docetaxel chemotherapy; Kaplan-Meier analysis followed by Gehan-Breslow-Wilcoxon test. **(C)** Western blot and the quantitative results showing the differential expression of PIGL protein between longer PFS and shorter PFS PCa biopsy tissues from mCRPC patients receiving docetaxel chemotherapy in our centers; unpaired two-tailed student’s t-test. **(D)** The association of expression level of PIGL protein with PFS and OS in PCa biopsy tissues from mCRPC patients receiving docetaxel chemotherapy; Kaplan-Meier analysis followed by Gehan-Breslow-Wilcoxon test. **(E)** RT-qPCR showing the differential expression of PIGL mRNA between docetaxel-resistant and docetaxel-sensitive PCa cells; unpaired two-tailed student’s t-test. **(F)** Western blot and the quantitative results showing the expression level of PIGL protein between docetaxel-resistant and docetaxel-sensitive PCa cells; unpaired two-tailed student’s t-test. ***P < 0.001.

Docetaxel was considered as the major chemotherapy drugs of PCa. We explored the role of the expression level of PIGL in the development of the docetaxel resistance of PCa. We conducted RT-qPCR to detect the mRNA expression level of PIGL in 20 cases of PCa biopsy tissues from mCRPC patients receiving docetaxel chemotherapy in our centers. According to the median progression-free survival (PFS) after receiving docetaxel chemotherapy, we divided all patients into longer PFS subgroup and shorter PFS subgroup. The results of RT-qPCR revealed that the difference of the mRNA expression level of PIGL between longer PFS subgroup and shorter PFS subgroup is not significant (**Figure 3A**). The data of survival revealed that the expression level of PIGL mRNA was not associated with PFS and overall survival (OS) in mCRPC patients receiving docetaxel chemotherapy in our centers (**Figure 3B**). We conducted western blot to detect the protein expression level of PIGL in 10 cases of PCa biopsy tissues from mCRPC patients receiving docetaxel chemotherapy in our centers. According to the median progression-free survival after receiving docetaxel chemotherapy, we divided all patients into longer PFS subgroup (P2, P4, P6, P8, P10) and shorter PFS subgroup (P1, P3, P5, P7, P9). The results of western blot revealed that the protein expression level of PIGL is highly increased in patients with shorter PFS compared with those with longer PFS (**Figure 3C**). The data of survival revealed that the high expression level of PIGL protein was associated with shorter PFS and OS in mCRPC patients receiving docetaxel chemotherapy in the First Affiliated Hospital of Fujian Medical University.

(**Figure 3D**). To explore the differential expression of PIGL between docetaxel-resistant and docetaxel-sensitive PCa cells, we constructed the docetaxel-resistant prostate cancer cells (named PC-3R and DU145R, respectively) using a method of gradient concentration increase of docetaxel in PC-3 and DU145. The results of the docetaxel IC50 value and colony formation assay under docetaxel treatment showed the successful construction of docetaxel-resistant prostate cancer cells (PC-3R and DU145R) (**Supplementary Figure 3**). The results of RT-qPCR showed that the differential expression of PIGL mRNA between docetaxel-resistant and docetaxel-sensitive PCa cells was not significant (**Figure 3E**). The results of western blot showed that the expression level of PIGL protein was significantly increased in docetaxel-resistant PCa cells compared with that in docetaxel-sensitive PCa cells (**Figure 3F**). These results showed that it was PIGL protein rather than mRNA that was expressed highly in docetaxel-resistant PCa. Hecne, the regulation of PIGL protein high expression in docetaxel-resistant PCa might be post-transcriptional level.

### High expression of PIGL promotes the docetaxel resistance of PCa

**Figure 4:**
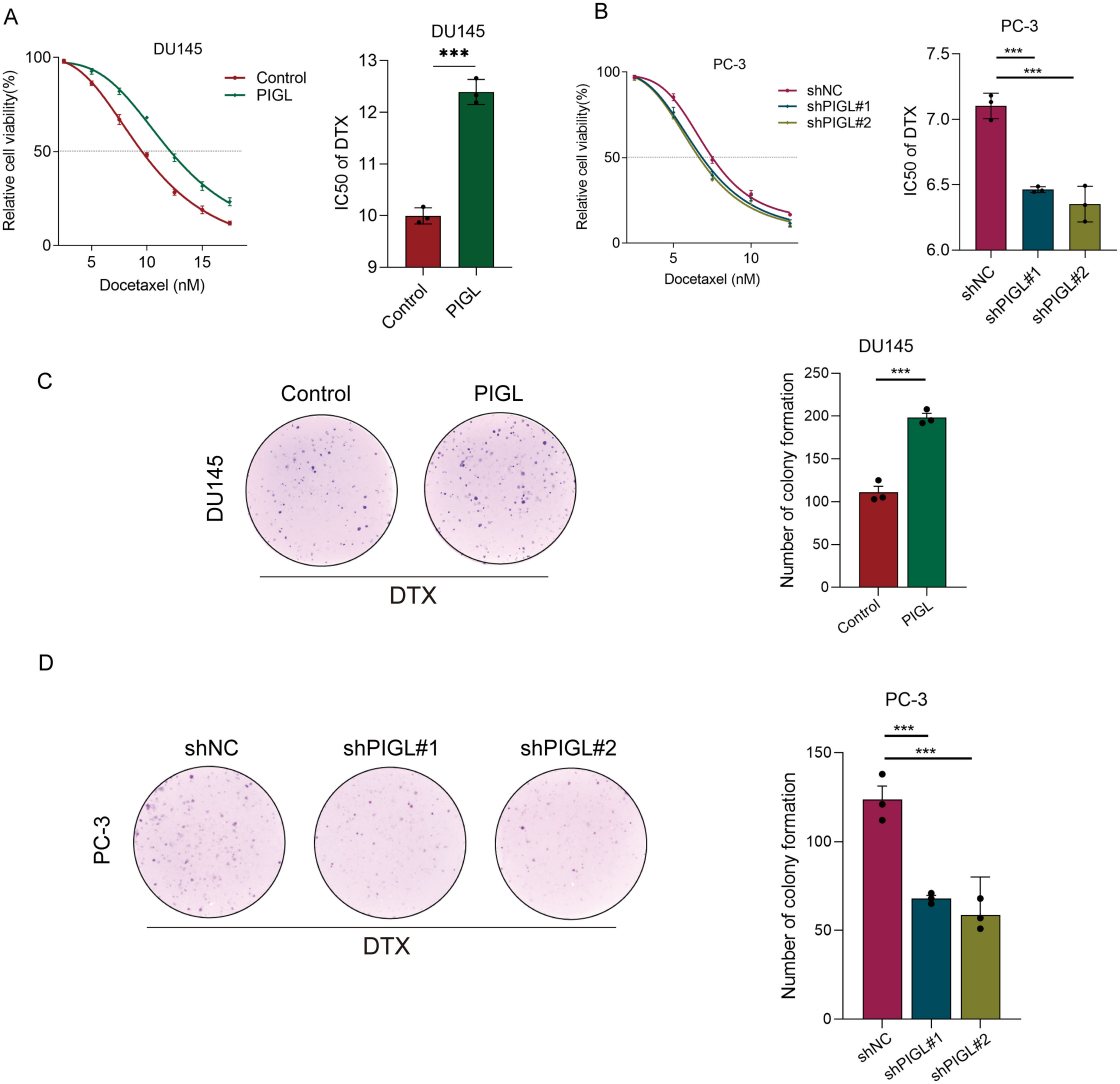
High expression of PIGL promotes the docetaxel resistance of PCa cells. **(A)** CCK8 toxicity assays showing that the effect of PIGL overexpression on the IC50 of docetaxel of DU145 cells; unpaired two-tailed student’s t-test. **(B)** CCK8 toxicity assays showing that the effect of PIGL knockdown on the IC50 of docetaxel of PC-3 cells; one-way ANOVA test. **(C)** Representative images and quantitative results of the colony formation assay showing the effect of PIGL overexpression on the clone formation capability of DU145 cells under docetaxel treatment; unpaired two-tailed student’s t-test. **(D)** Representative images and quantitative results of the colony formation assay showing the effect of PIGL knockdown on the clone formation capability of PC-3 cells under docetaxel treatment; one-way ANOVA test. ***P < 0.001.

The results of CCK8 toxicity assays demonstrated that the overexpression of PIGL elevated the IC50 of docetaxel of DU145 cells and the knockdown of PIGL reduced the IC50 of docetaxel of PC-3 cells (**Figures 4A, B**). The results of colony formation assay showed that the overexpression of PIGL promotes the clone formation capability of DU145 cells under docetaxel treatment and the knockdown of PIGL inhibits the clone formation capability of PC-3 cells under docetaxel treatment (**Figures 4C, D**). These results showed that high expression of PIGL promotes the docetaxel resistance of PCa.

### Identification of potential binding proteins of PIGL in PCa

**Figure 5:**
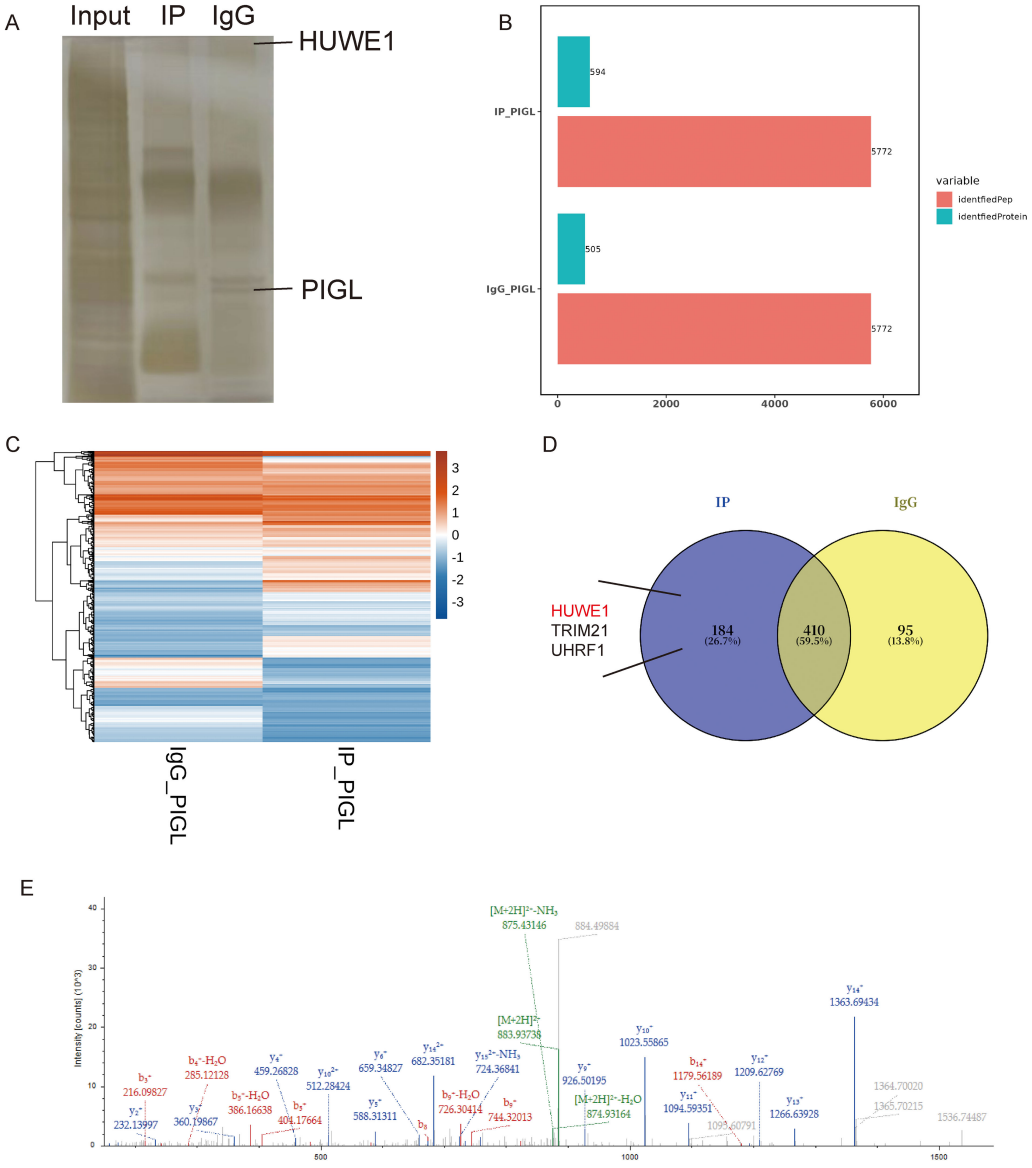
Identification of the potential binding proteins of PIGL in PCa cells using IP-MS. **(A–E)** Mass spectrometry after immunoprecipitation by anti-Flag antibody using lysates from DU145 cells transfected with Flag-PIGL identifying the binding proteins of PIGL, and there were three potential E3 ubiquitin ligase, including HUWE1, TRIM21, UHRF1, of which HUWE1 has a highest score.

**Figure 6:**
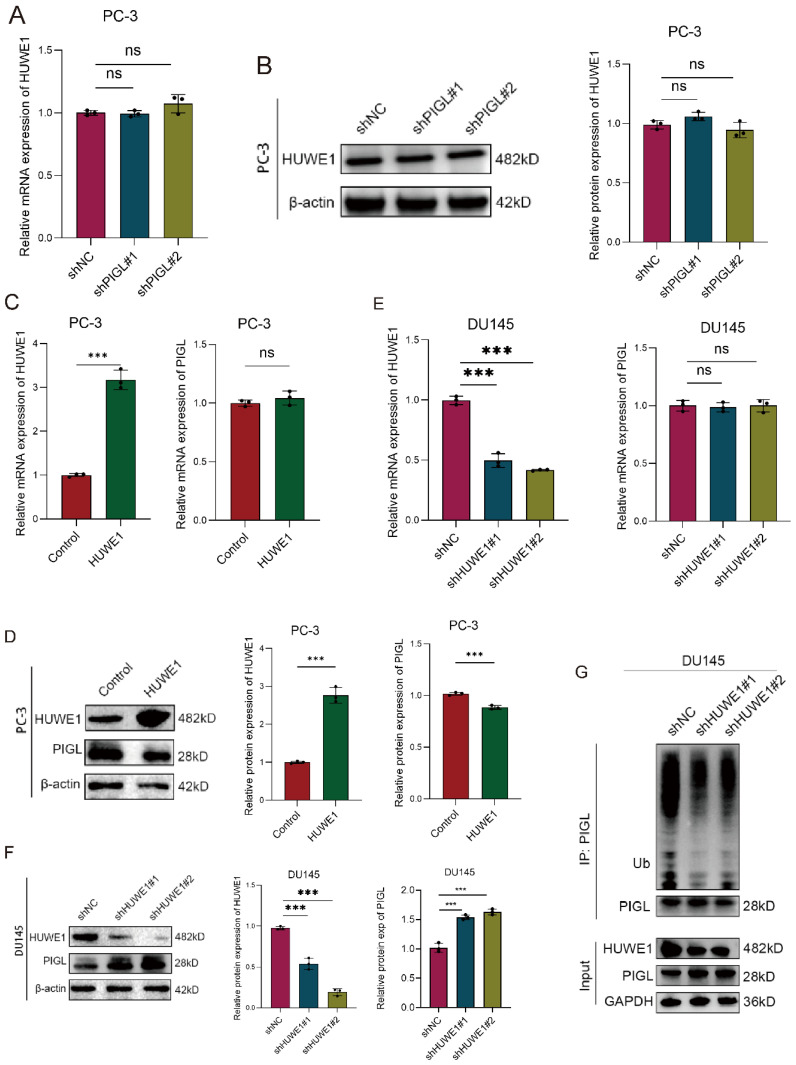
HUWE1 regulates the expression of PIGL protein rather than mRNA. **(A)** RT-qPCR showing the effect PIGL silencing on the mRNA expression of HUWE1 in PC-3 cells; one-way ANOVA test. **(B)** Western blot and the quantitative results showing the effect PIGL silencing on the protein expression of HUWE1 in PC-3 cells; one-way ANOVA test. **(C)** RT-qPCR indicating the mRNA efficiency of HUWE1 overexpression in PC-3 cells and the effect HUWE1 overexpression on the mRNA expression of PIGL in PC-3 cells; unpaired two-tailed student’s t-test. **(D)** Western blot and the quantitative results showing the protein efficiency of HUWE1 overexpression in PC-3 cells and the effect HUWE1 overexpression on the protein expression of PIGL in PC-3 cells; unpaired two-tailed student’s t-test **(E)** RT-qPCR indicating the mRNA efficiency of HUWE1 knockdown in DU145 cells and the effect of HUWE1 knockdown on the mRNA expression of PIGL in DU145 cells; one-way ANOVA test. **(F)** Western blot and the quantitative results showing the protein efficiency of HUWE1 knockdown in DU145 cells and the effect HUWE1 knockdown on the protein expression of PIGL inDU145 cells; one-way ANOVA test. **(G)** Endogenous ubiquitination assay showing the effect of HUWE1 knockdown on the poly-ubiquitination of PIGL in CRPC cells treated with MG132 (10 μM) for 6 h. ***P < 0.001.

For the reason that the expression levels of PIGL protein rather than mRNA was upregulated in docetaxel-resistant PCa cells and associated with the PFS of patients receiving docetaxel chemotherapy, our study initiates an investigation into the landscape of posttranslational regulation. Ubiquitination is among the most common post-translational modifications governing the precise degradation of cellular proteins (13); hence, whole-cell extracts from DU145 cells transfected with Flag-PIGL were applied to identify binding ubiquitination-related partners of the PIGL protein and test potential posttranslational mechanisms implicated in the regulation of PIGL protein expression. After electrophoretic separation on SDS–PAGE gels, the protein products were visualized by silver staining and analyzed using mass spectrometry (**Figures 5A–C**). Several ubiquitination-related proteins, including HUWE1, TRIM21 and UHRE1, were identified as potential interacting partners of PIGL, of which HUWE1 has a highest binding score (**Figure 5D**). The peptide spectrogram of HUWE1 is shown in **Figure 5E**. The results of RT-qPCR and western showed that the PIGL silencing did not affect the mRNA and protein expression of HUWE1 in PC-3 cells, which indicated that PIGL is not the upstream of HUWE1 (**Figures 6A, B**). Next, to investigate whether HUWE1 could regulate the expression of PIGL, we constructed the stable PCa cell lines with HUWE1 overexpression or knockdown. The results of RT-qPCR and western showed that the HUWE1 overexpression results in the low expression of PIGL protein rather than mRNA in PC-3 cells (**Figures 6C, D**). The results of RT-qPCR and western showed that the HUWE1 silencing results in the high expression of PIGL protein rather than mRNA in DU145 cells (**Figures 6E, F**). The results of co-immunoprecipitation assay followed by western blot showed that PIGL protein could directly bind to HUWE1 protein in DU145 cells (**Supplementary Figure 4A**). Next, we explored whether HUWE1 could increase the conjugated ubiquitin of PIGL protein. The results of endogenous ubiquitination assay showed that the polyubiquitination level of PIGL was markedly decreased after HUWE1 knockdown in DU145 cells (**Figure 6G**). Western blot analysis showed that MG132 rather than CQ treatment increased the PIGL protein expression level in DU145 cells. Hence, PIGL protein is degraded through proteasome pathway (**Supplementary Figure 4B**). These results showed that the E3 ubiquitin ligase HUWE1 is the upstream of PIGL and HUWE1 could negatively regulate the expression of PIGL in PCa cells.

### HUWE1 was downregulated in docetaxel-resistant PCa

**Figure 7:**
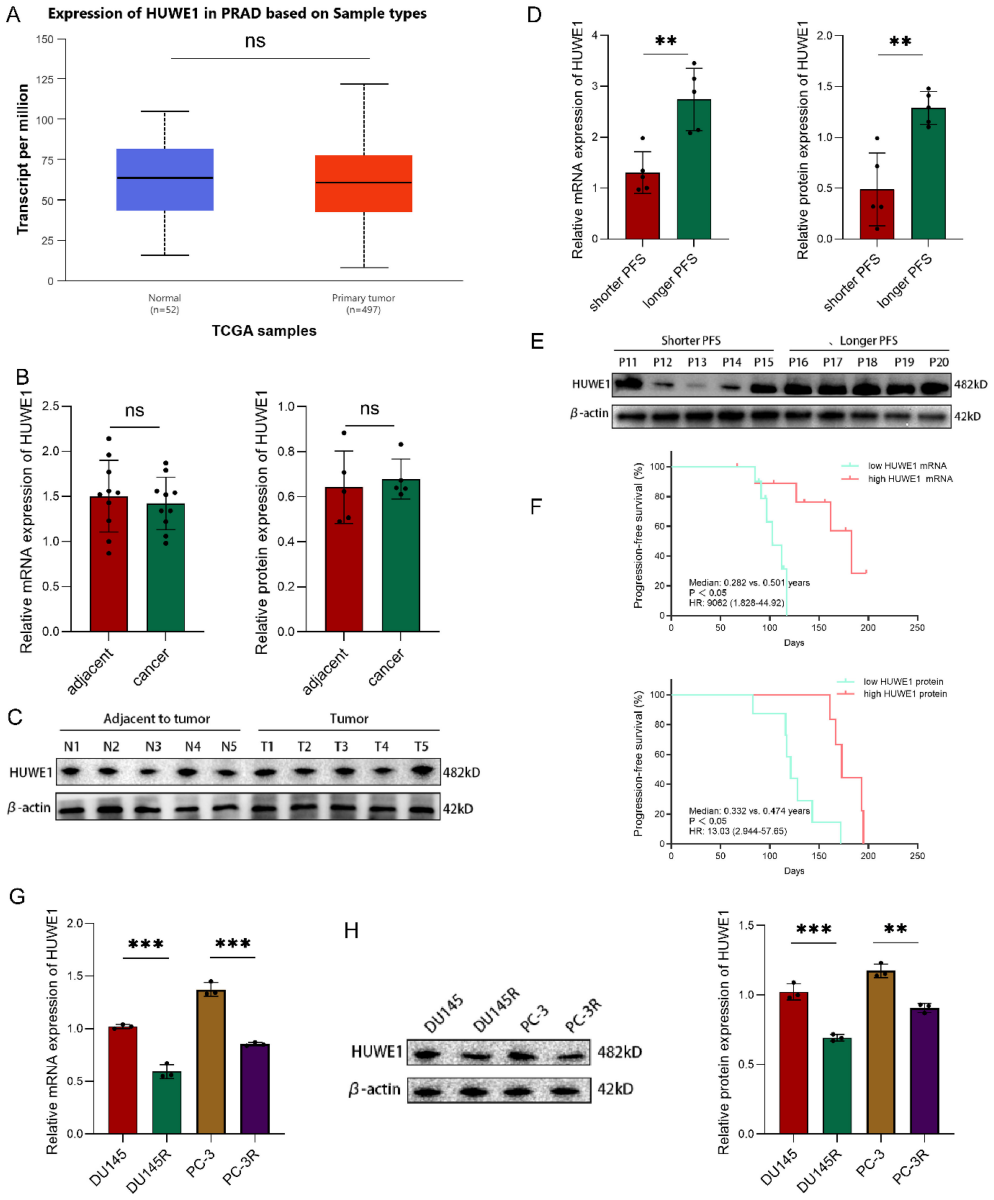
HUWE1 was downregulated in docetaxel-resistant PCa cells and tissues and associated with prognosis. **(A)** The differential expression of HUWE1 mRNA between normal and PCa tissues analyzed by TCGA-PARD dataset. **(B)** RT-qPCR showing the differential expression of HUWE1 mRNA between PCa tissues and paired tissues adjacent to cancer from patients in our centers; paired two-tailed student’s t-test. **(C)** Western blot and the quantitative results showing the differential expression of HUWE1 protein between PCa tissues and paired tissues adjacent to cancer from patients in our centers; paired two-tailed student’s t-test. **(D)** RT-qPCR showing the differential expression of HUWE1 mRNA between longer PFS and shorter PFS PCa biopsy tissues from mCRPC patients receiving docetaxel chemotherapy in our centers; unpaired two-tailed student’s t-test. **(E)** Western blot and the quantitative results showing the differential expression of HUWE1 protein between longer PFS and shorter PFS PCa biopsy tissues from mCRPC patients receiving docetaxel chemotherapy in our centers; unpaired two-tailed student’s t-test. **(F)** The association of expression level of HUWE1 mRNA and protein with PFS in PCa biopsy tissues from mCRPC patients receiving docetaxel chemotherapy. **(G)** RT-qPCR showing the differential expression of HUWE1 mRNA between docetaxel-resistant and docetaxel-sensitive PCa cells; unpaired two-tailed student’s t-test. **(H)** Western blot and the quantitative results showing the expression level of HUWE1 protein between docetaxel-resistant and docetaxel-sensitive PCa cells; unpaired two-tailed student’s t-test. **P < 0.01, ***P < 0.001.

We analyzed the data from TCGA-PARD dataset and found that the mRNA expression level of HUWE1 between PCa tissues and normal tissues was not significant (**Figure 7A**). We conducted RT-qPCR to detect the mRNA expression level of HUWE1 in 10 cases of PCa tissues and paired tissues adjacent to cancer in our centers, which revealed that the mRNA expression level of HUWE1 between PCa tissues and paired tissues adjacent to cancer was not significant (**Figure 7B**). We conducted western blot to detect the protein expression level of HUWE1 in 5 cases of PCa tissues and paired tissues adjacent to cancer in our centers, which revealed that protein expression level of HUWE1 between PCa tissues and paired tissues adjacent to cancer was not significant (**Figure 7C**).

Next, we conducted RT-qPCR and western blot to detect the mRNA and protein expression level of HUWE1 in 10 cases of PCa biopsy tissues from mCRPC patients receiving docetaxel chemotherapy in our centers. According to the median progression-free survival (PFS) after receiving docetaxel chemotherapy, we divided all patients into longer PFS subgroup and shorter PFS subgroup. The results of RT-qPCR and western blot revealed that the mRNA and protein expression level of HUWE1 was significantly decreased in shorter PFS subgroup compared with longer PFS subgroup (**Figures 7D, E**). The data of survival analysis revealed that the low expression level of HUWE1 mRNA and protein was associated with poor PFS in mCRPC patients receiving docetaxel chemotherapy in our centers (**Figure 7F**). Besides, another independent validation cohort of Second People’s Hospital of Yichang also showed that the low expression level of HUWE1 mRNA and protein was associated with poor PFS in mCRPC patients receiving docetaxel chemotherapy (**Supplementary Figure 5**). We also explored the differential expression of HUWE1 mRNA and protein between docetaxel-resistant and docetaxel-sensitive PCa cells. The results of RT-qPCR and western blot showed that the expression level of HUWE1 was significantly decreased in docetaxel-resistant PCa cells compared with that in docetaxel-sensitive PCa cells (**Figures 7G, H**). These results showed that the expression level of HUWE1 protein and mRNA was significantly decreased in docetaxel-resistant PCa.

### Downregulation of HUWE1 promotes the docetaxel resistance of PCa via elevating PIGL

**Figure 8:**
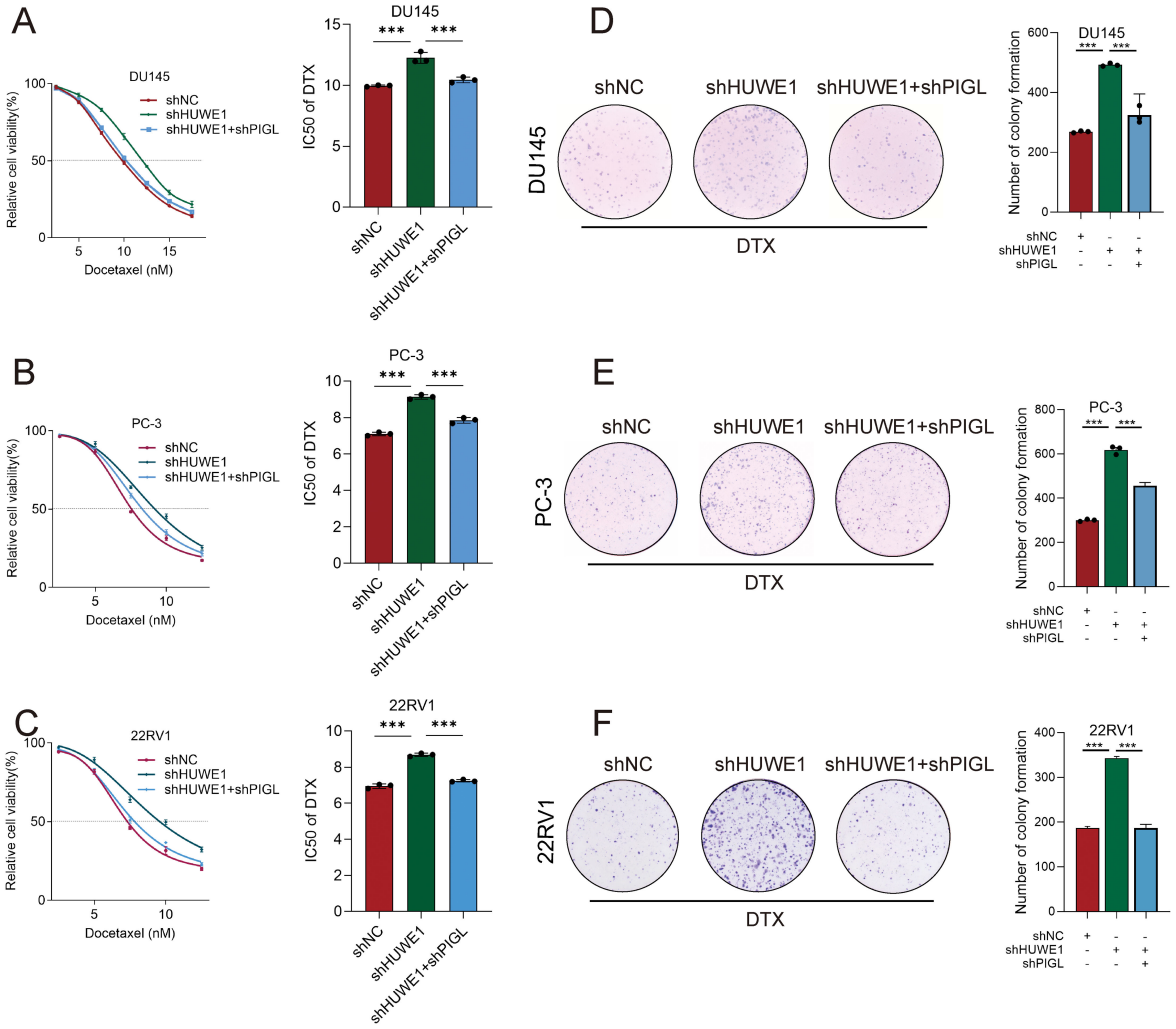
Low expression of HUWE1 promotes the docetaxel resistance of PCa cells via upregulating PIGL. **(A–C)** CCK8 toxicity assays showed that the effect of HUWE1 knockdown on the IC50 of docetaxel of DU145, PC-3, and 22RV1 cells could be rescued by PIGL silencing; two-way repeated measures ANOVA test. **(D–F)** Representative images and quantitative results of the colony formation assay showed that the effect of HUWE1 knockdown on the clone formation capability of DU145, PC-3, and 22RV1 cells under docetaxel treatment could be rescued by PIGL silencing; one-way ANOVA test. ***P < 0.001.

The results of CCK8 toxicity assays demonstrated that the knockdown of HUWE1 elevated the IC50 of docetaxel of DU145, PC-3, 22RV1 cells and could be rescued by PIGL silencing (**Figures 8A–C**). The results of colony formation assay showed that the knockdown of HUWE1 promotes the clone formation capability of DU145, PC-3, 22RV1 cells under docetaxel treatment and could be rescued by PIGL silencing (**Figures 8D–F**). The results of flow cytometry apoptosis assay demonstrated that the knockdown of HUWE1 inhibited the apoptosis of DU145 and PC-3 cells under docetaxel treatment and could be rescued by knockdown of PIGL (**Supplementary Figure 6**). These results showed that downregulation of HUWE1 promotes the docetaxel resistance of PCa via elevating PIGL.

## Discussion

PCa is considered as a heterogeneous malignant tumor and is curable in early stages; however, the prognosis is rather poor and the treatment is rather difficult at advanced stages (14). The challenges in treating PCa of late stage includes immunosuppressive microenvironment, high genomic heterogeneity and instability, androgen-independence mechanisms, and so on (15). Therapies would result in treatment resistance mechanisms in PCa cells. Radiotherapy would stimulate the damage of DNA and the generation of ROS to kill PCa cell; however, ROS generation and DNA damage would lead to antioxidant defense mechanism, which results in radio-resistance (16). Inhibition of sphingosine kinase could increase the sensitivity to enzalutamide of PCa cells (17). The down-regulation of NXTAR would activate AR signaling and promote the resistance to enzalutamide of PCa cells (18). Chemotherapy using docetaxel was regarded as the major treatment in PCa from palliation to improving overall survival (19). In PCa of advanced stage, docetaxel-based chemotherapy was widely used in metastatic hormone sensitive prostate cancer and metastatic castration-resistant prostate cancer (20). Besides, docetaxel-based neoadjuvant chemotherapy was also used in numerous clinical trials (21). However, as far as we know, docetaxel resistance was more common in PCa than other cancers (22), and the specific molecular mechanisms are not fully illuminated (23).

There were many studies investigating the potential molecular mechanism of docetaxel resistance in PCa. Ge, et al. reported that ASPN is highly expressed in PCa and is activated by TGFβ and interacts with STMN1, enhancing the stemness and epithelial-mesenchymal transition by activating Wnt/β-catenin pathway, finally promoting the docetaxel resistance of PCa (24). Wang, et al. revealed that AGD1 plays an important role in promoting resistance to docetaxel by USP10/METTL13-mediated CD44 mRNA decay in castration resistant PCa (25). Li, et al. demonstrated that USP3 directly interacts with SMARCA5 and removes the polyubiquitination of SMARCA5, thereby promoting the docetaxel resistance of PCa (26). Ying, et al. revealed that IGF2BP proteins regulated the R-loop resolution by an m6A-dependent way, which could result in docetaxel chemotherapy resistance of PCa (27). Gomes, et al. found that it was p-AKT protein rather than PTEN that could predict response to docetaxel combined with AKT inhibition (21). Androgen receptor also functions in the development of docetaxel resistance. Komura, et al (28). found that PCa docetaxel resistance is associated with androgen receptor activation and loss of KDM5D expression. Lu, et al (22). revealed that docetaxel-resistant subclones cells had stronger activation of androgen receptor and PI3K/Akt pathway and quercetin reversed docetaxel resistance in prostate cancer via androgen receptor. Phosphatidylinositol Glycan Anchor Biosynthesis Class L (PIGL) was an enzyme that catalyzes the second step of glycosylphosphatidylinositol (GPI) biosynthesis, which is the de-N-acetylation of N-acetylglucosaminylphosphatidylinositol (GlcNAc-PI) (29). There are very few studies exploring the role of PIGL in the occurrence and development of cancers. Yu, et al (11). found that the intracellular sub-localization of PIGL might be a vital factor driving tumor immune evasion. They revealed that Y81 of PIGL could be phosphorylated by FGFR2 to abolish the interaction of with importin α/β1, thus retaining PIGL in the cytoplasm and promoting immune evasion by releasing CCL2 and CCL20. However, no study investigating the effect of PIGL in the PCa development and docetaxel resistance. In our study, we demonstrated that both the mRNA and protein of PIGL is highly expressed in prostate cancer (PCa) and associated with poor prognosis. More importantly, we found that the protein rather than mRNA of PIGL is highly expressed in docetaxel-resistant PCa tissues and could predict the prognosis of PCa receiving docetaxel chemotherapy. Our results showed that high expression level of PIGL promotes the proliferation, migration, invasion, and docetaxel resistance of PCa. These findings emphasized the vital role of PIGL in the progression and docetaxel resistance of PCa and would contribute to the potential mechanism of resistance to docetaxel chemotherapy in PCa patients.

Our results showed that it was PIGL protein rather than mRNA that was expressed highly in docetaxel-resistant PCa, which revealed that the regulation of PIGL protein high expression might be post-transcriptional level. To explore the mechanism of PIGL protein high expression, we used the mass spectrometry to identify the potential binding proteins of PIGL. It was found that the E3 ubiquitin ligase HUWE1 is the upstream of PIGL and HUWE1 could negatively regulate the expression of PIGL in PCa cells. HECT, UBA And WWE Domain Containing E3 Ubiquitin Protein Ligase 1 (HUWE1) is a E3 ubiquitin-protein ligase which mediates ubiquitination and subsequent proteasomal degradation of target proteins (30). There were several studies exploring the role of HUWE1 in PCa. Lin, et al (31). revealed that R1 regulated the transcriptional suppression of HUWE1 to increases protein stability of c-Myc by inhibiting ubiquitination and proteolysis and then regulates PCa growth and progression. Qu, et al (32). also found that the upregulation of HUWE1 has tumor suppressive effect of PCa via downregulating c-Myc. Fan, et al (33). revealed that JMJD1A bound to HUWE1 and then attenuated HUWE1-dependent ubiquitination to increase the protein levels of c-Myc to regulate the PCa growth and survival. Fan, et al (34). demonstrated that HUWE1 plays a vital role in inducing the K27-/K29-linked noncanonical ubiquitination of JMJD1A and then regulates the radio-resistance of PCa. However, no study investigating the effect of HUWE1 in the PCa docetaxel resistance and the regulated role of HUWE1 and PIGL. In our study, we firstly found that the expression level of HUWE1 protein and mRNA was significantly decreased in docetaxel-resistant PCa, and the downregulation of HUWE1 promotes the docetaxel resistance of PCa via elevating PIGL. These findings emphasized the vital role of HUWE1/PIGL axis in the docetaxel resistance of PCa and the effect of the E3 ubiquitin ligase HUWE1 on regulating the protein expression of PIGL.

There were several limitations of this study. Firstly, although we revealed the role of PIGL in the progression and docetaxel resistance of PCa using bioinformatics analysis and *in vitro* study, absence of immune-related *in vivo* data is the major limitation of our study and required further validation in future. Secondly, the underlying regulatory mechanism of the downstream of PIGL remains uncertain. Further studies will be needed to elucidate the underlying regulatory mechanism.

## Conclusion

Both the mRNA and protein of PIGL is highly expressed in PCa and associated with poor prognosis. The protein rather than mRNA of PIGL is highly expressed in docetaxel-resistant PCa tissues and could predict the prognosis of PCa receiving docetaxel chemotherapy. High expression of PIGL promotes the tumor formation and docetaxel resistance of PCa. The downregulation of HUWE1 promotes the docetaxel resistance of PCa via elevating PIGL. These findings emphasized the vital role of HUWE1/PIGL axis in the docetaxel resistance of PCa and the effect of the E3 ubiquitin ligase HUWE1 on regulating the protein expression of PIGL.

## Data Availability

The original contributions presented in the study are included in the article/**Supplementary Material**. Further inquiries can be directed to the corresponding authors.

## Supplementary material

Supplementary Figure 1 The baseline expression and the efficiency of PIGL overexpression or knockdown in PCa cell lines. **(A)** RT-qPCR showing the expression level of PIGL mRNA in different PCa cells. **(B)** Western blot and the quantitative results showing the expression level of PIGL protein in different PCa cells. **(C)** RT-qPCR indicating the mRNA efficiency of PIGL overexpression in DU145 cells and PIGL knockdown in PC-3 cells; one-way ANOVA test and unpaired two-tailed student’s t-test. **(D)** Western blot indicating the protein efficiency of PIGL overexpression in DU145 cells and PIGL knockdown in PC-3 cells; one-way ANOVA test and unpaired two-tailed student’s t-test. **P* < 0.05, ***P* < 0.01, ****P* < 0.001.

Supplementary Figure 2 The effect of PIGL on the expression of CCL2 and CCL20 in PCa cells. **(A)** The effect of PIGL on the expression of CCL2 and CCL20 mRNA in PCa cells.; unpaired two-tailed student’s t-test or one-way ANOVA test. **(B)** The effect of PIGL on the expression of CCL2 and CCL20 content in the culture medium of PCa cells.; unpaired two-tailed student’s t-test or one-way ANOVA test.

Supplementary Figure 3 Validation of the successful construction of docetaxel-resistant prostate cancer cells. **(A)** CCK8 toxicity assays showing the difference of the docetaxel IC50 between docetaxel-resistant and docetaxel-sensitive prostate cancer cells (PC-3R vs. PC-3; DU145R vs, DU145); unpaired two-tailed student’s t-test. **(B)** Representative images and quantitative results of cloning formation assay showing the difference of the clone formation capability between docetaxel-resistant and docetaxel-sensitive prostate cancer cells (PC-3R vs. PC-3; DU145R vs, DU145); unpaired two-tailed student’s t-test. **P* < 0.05, ***P* < 0.01, ****P* < 0.001.

Supplementary Figure 4 The combination of PIGL and HUWE1 and the degradation pathway of PIGL protein. **(A)** co-immunoprecipitation assay followed by western blot showing the combination of PIGL protein and HUWE1 protein. **(B)** Western blot showing the expression levels of PIGL protein after treating with CQ or MG132; one-way ANOVA test. **P* < 0.05, ***P* < 0.01, ****P* < 0.001.

Supplementary Figure 5 The association of expression level of HUWE1 mRNA and protein with PFS in PCa biopsy tissues from mCRPC patients receiving docetaxel chemotherapy from another independent validation cohort of Second People’s Hospital of Yichang.

Supplementary Figure 6 Low expression of HUWE1 inhibits the apoptosis of PCa cells under docetaxel treatment. Flow cytometry apoptosis assay showed that the effect of HUWE1 knockdown on the apoptosis under docetaxel treatment of DU145 and PC-3 cells could be rescued by PIGL silencing; one-way ANOVA test. **P* < 0.05, ***P* < 0.01, ****P* < 0.001.
