# Correction: PIGL promotes the docetaxel resistance of prostate cancer and is regulated by E3 ubiquitin ligase HUWE1

**DOI:** 10.3389/fimmu.2026.1933318

**Published:** 2026-07-28

**Authors:** Pan Gao, Bo-Han Lin, Zhi Zhang, Ze-Yu Yi, Zhi-Bin Ke, Zhong-Hua Zhu, Zhen Kang, Yong Wei

**Affiliations:** 1 Department of Urology, Urology Research Institute, The First Affiliated Hospital, Fujian Medical University, Fuzhou, China; 2 Department of Urology, National Regional Medical Center, Binhai Campus of the First Affiliated Hospital, Fujian Medical University, Fuzhou, China; 3 Department of Urology, Second People’s Hospital of Yichang, Yichang, China; 4 Department of Urology, Second People’s Hospital of China Three Gorges University, Yichang, China; 5 Department of Urology, The First People’s Hospital of Yunnan Province, Kunming, China; 6 Department of Urology, The Affiliated Hospital of Kunming University of Science and Technology, Kunming, China

**Keywords:** docetaxel resistance, HUWE1 gene, PIGL gene, prostate cancer, ubiquitination

There was a mistake in **Figure 1**, **Figure 3**, **Figure 6**, **Figure 7** as published. All Western blot panels in the **Figure 1**, **Figure 3**, **Figure 6**, and **Figure 7** have been incorrectly replaced with the original gel images, and all group information, condition annotations, and molecular weight markers were erroneously removed. The corrected **Figure 1**, **Figure 3**, **Figure 6**, and **Figure 7** appears below.

**Figure 1:**
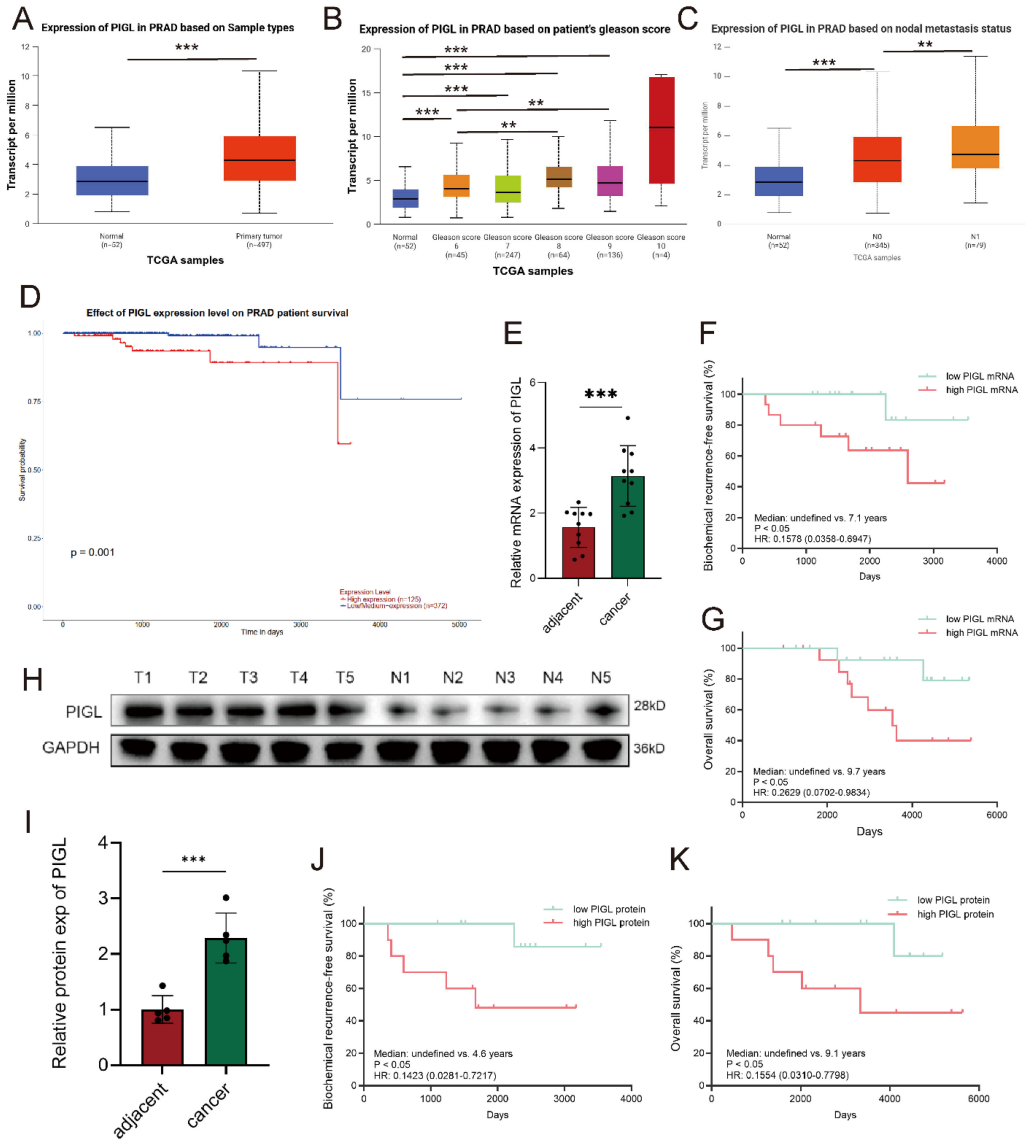
PIGL is highly expressed in PCa and associated with poor prognosis. **(A)** The differential expression of PIGL mRNA between normal and PCa tissues analyzed by TCGA-PARD dataset. **(B)** The association of expression level of PIGL mRNA with Gleason score analyzed by TCGA-PARD dataset. **(C)** The association of expression level of PIGL mRNA with nodal metastasis analyzed by TCGA-PARD dataset. **(D)** The association of expression level of PIGL mRNA with overall survival (OS) analyzed by TCGA PARD dataset. **(E)** RT-qPCR showing the differential expression of PIGL mRNA between PCa tissues and paired tissues adjacent to cancer from patients in our centers; paired two-tailed student’s t-test. **(F, G)** The association of expression level of PIGL mRNA in PCa tissues from patients in our centers with biochemical recurrence-free survival (BCRFS) and OS; Kaplan-Meier analysis Gehan-Breslow-Wilcoxon test. **(H, I)** Western blot and the quantitative results showing the differential expression of PIGL protein between PCa tissues and paired tissues adjacent to cancer from patients in our centers; paired two-tailed student’s t-test. **(J, K)** The association of expression level of PIGL protein in PCa tissues from patients in our centers with BCRFS and OS; Kaplan-Meier analysis followed by Gehan-Breslow-Wilcoxon test. **P < 0.01, ***P < 0.001.

**Figure 3:**
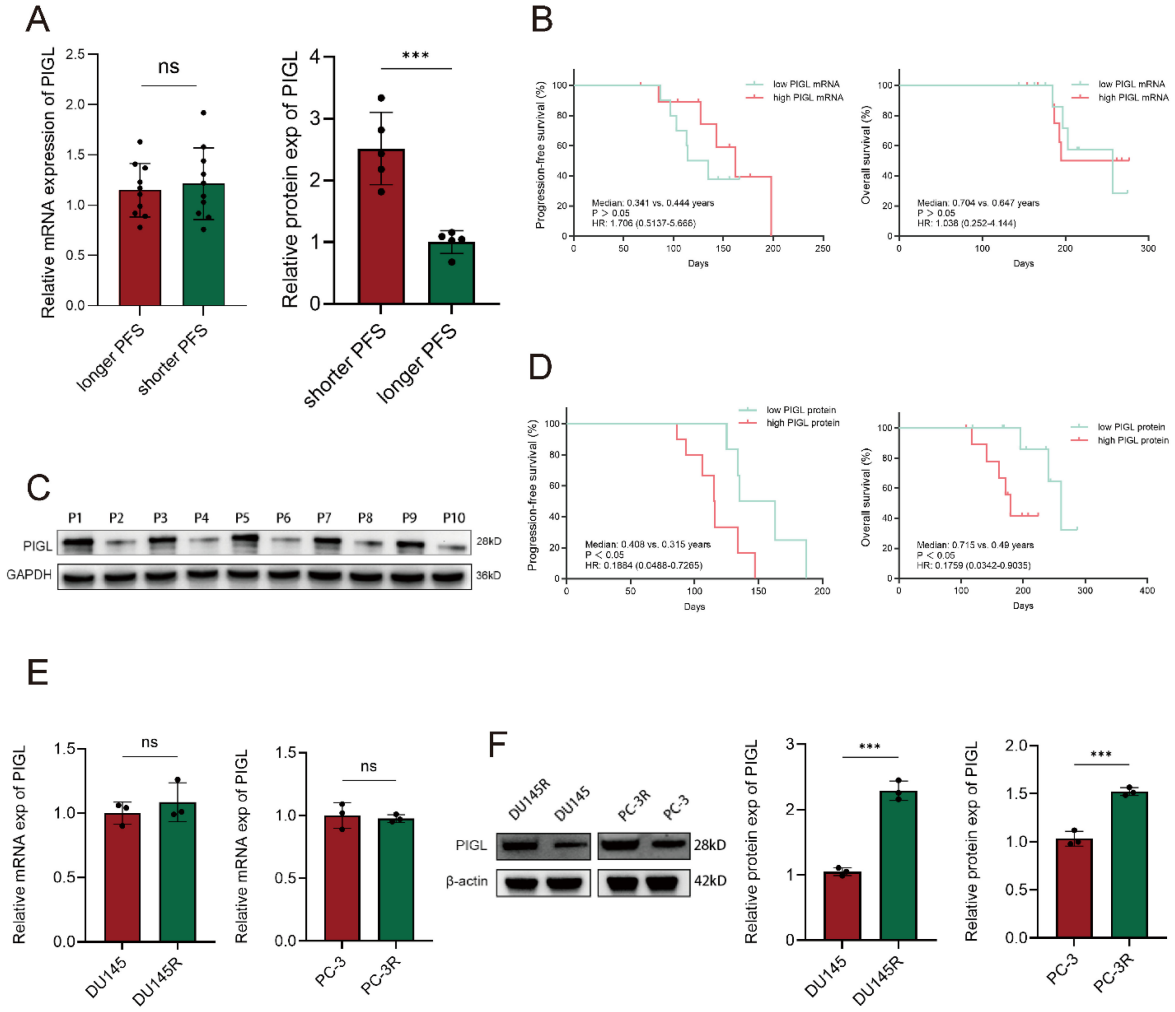
The expression level of PIGL protein rather than mRNA was increased in the docetaxel-resistant PCa cells and tissues. **(A)** RT-qPCR showing the differential expression of PIGL mRNA between longer progression-free survival (PFS) and shorter PFS PCa biopsy tissues from mCRPC patients receiving docetaxel chemotherapy in our centers; unpaired two-tailed student’s t-test. **(B)** The association of expression level of PIGL mRNA with PFS and overall survival (OS) in PCa biopsy tissues from mCRPC patients receiving docetaxel chemotherapy; Kaplan-Meier analysis followed by Gehan-Breslow-Wilcoxon test. **(C)** Western blot and the quantitative results showing the differential expression of PIGL protein between longer PFS and shorter PFS PCa biopsy tissues from mCRPC patients receiving docetaxel chemotherapy in our centers; unpaired two-tailed student’s t-test. **(D)** The association of expression level of PIGL protein with PFS and OS in PCa biopsy tissues from mCRPC patients receiving docetaxel chemotherapy; Kaplan-Meier analysis followed by Gehan-Breslow-Wilcoxon test. **(E)** RT-qPCR showing the differential expression of PIGL mRNA between docetaxel-resistant and docetaxel-sensitive PCa cells; unpaired two-tailed student’s t-test. **(F)** Western blot and the quantitative results showing the expression level of PIGL protein between docetaxel-resistant and docetaxel-sensitive PCa cells; unpaired two-tailed student’s t-test. ***P < 0.001.

**Figure 6:**
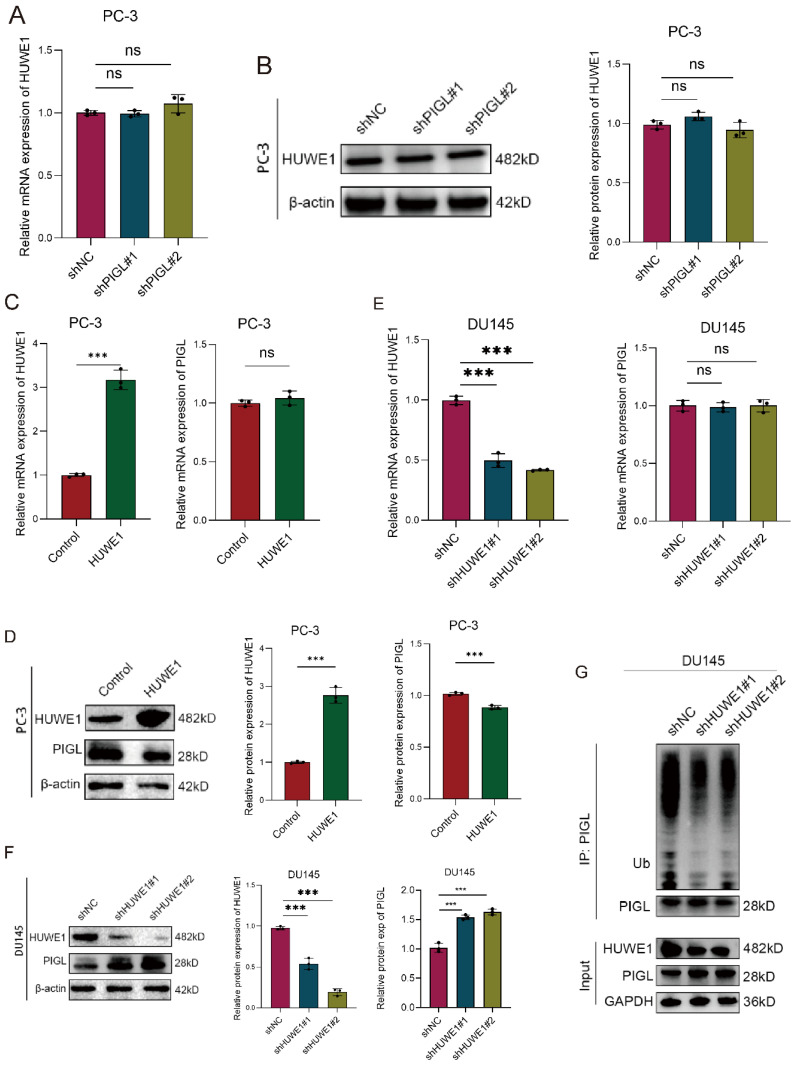
HUWE1 regulates the expression of PIGL protein rather than mRNA. **(A)** RT-qPCR showing the effect PIGL silencing on the mRNA expression of HUWE1 in PC-3 cells; one-way ANOVA test. **(B)** Western blot and the quantitative results showing the effect PIGL silencing on the protein expression of HUWE1 in PC-3 cells; one-way ANOVA test. **(C)** RT-qPCR indicating the mRNA efficiency of HUWE1 overexpression in PC-3 cells and the effect HUWE1 overexpression on the mRNA expression of PIGL in PC-3 cells; unpaired two-tailed student’s t-test. **(D)** Western blot and the quantitative results showing the protein efficiency of HUWE1 overexpression in PC-3 cells and the effect HUWE1 overexpression on the protein expression of PIGL in PC-3 cells; unpaired two-tailed student’s t-test **(E)** RT-qPCR indicating the mRNA efficiency of HUWE1 knockdown in DU145 cells and the effect of HUWE1 knockdown on the mRNA expression of PIGL in DU145 cells; one-way ANOVA test. **(F)** Western blot and the quantitative results showing the protein efficiency of HUWE1 knockdown in DU145 cells and the effect HUWE1 knockdown on the protein expression of PIGL inDU145 cells; one-way ANOVA test. **(G)** Endogenous ubiquitination assay showing the effect of HUWE1 knockdown on the poly-ubiquitination of PIGL in CRPC cells treated with MG132 (10 μM) for 6 h. ***P < 0.001.

**Figure 7:**
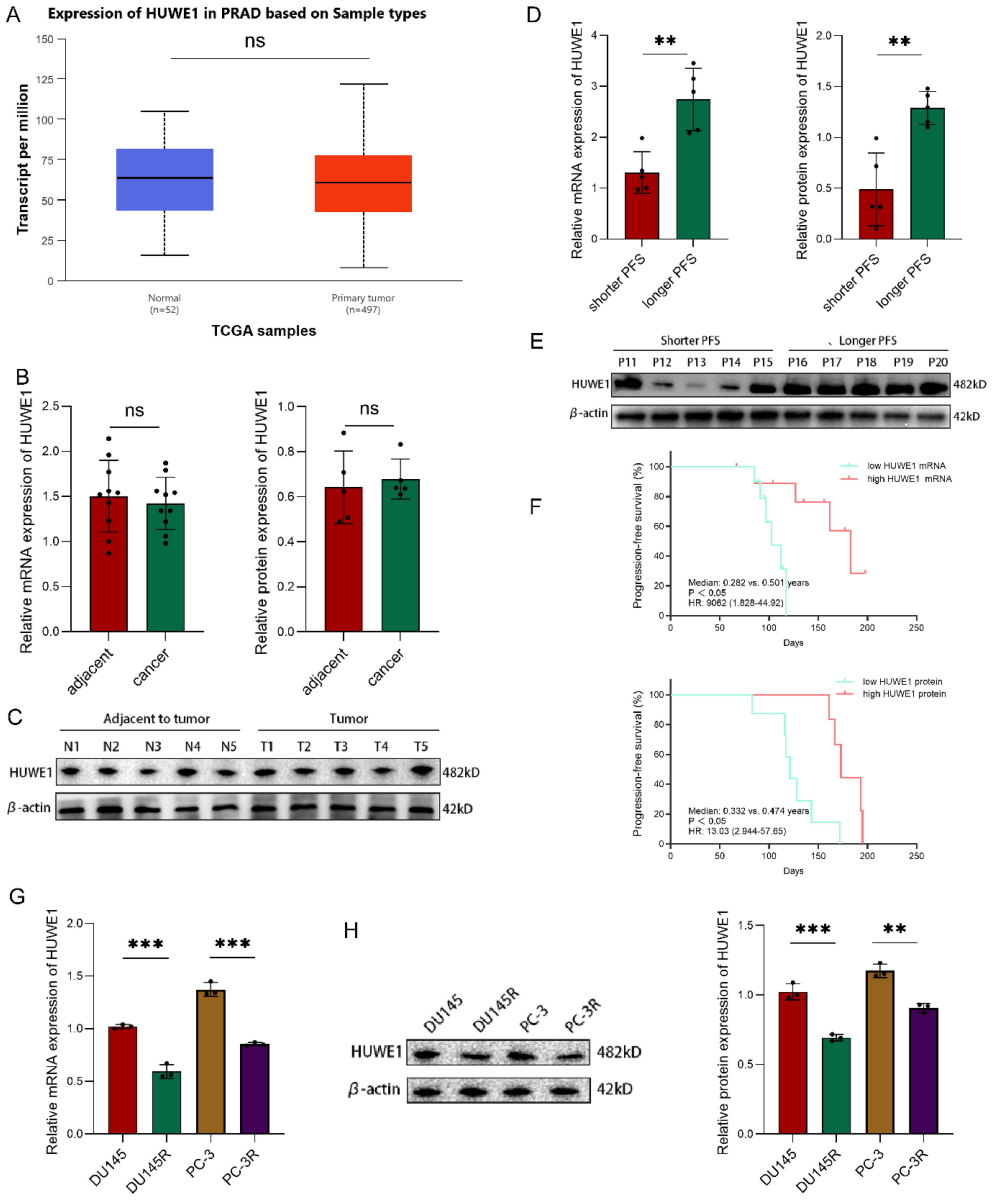
HUWE1 was downregulated in docetaxel-resistant PCa cells and tissues and associated with prognosis. **(A)** The differential expression of HUWE1 mRNA between normal and PCa tissues analyzed by TCGA-PARD dataset. **(B)** RT-qPCR showing the differential expression of HUWE1 mRNA between PCa tissues and paired tissues adjacent to cancer from patients in our centers; paired two-tailed student’s t-test. **(C)** Western blot and the quantitative results showing the differential expression of HUWE1 protein between PCa tissues and paired tissues adjacent to cancer from patients in our centers; paired two-tailed student’s t-test. **(D)** RT-qPCR showing the differential expression of HUWE1 mRNA between longer PFS and shorter PFS PCa biopsy tissues from mCRPC patients receiving docetaxel chemotherapy in our centers; unpaired two-tailed student’s t-test. **(E)** Western blot and the quantitative results showing the differential expression of HUWE1 protein between longer PFS and shorter PFS PCa biopsy tissues from mCRPC patients receiving docetaxel chemotherapy in our centers; unpaired two-tailed student’s t-test. **(F)** The association of expression level of HUWE1 mRNA and protein with PFS in PCa biopsy tissues from mCRPC patients receiving docetaxel chemotherapy. **(G)** RT-qPCR showing the differential expression of HUWE1 mRNA between docetaxel-resistant and docetaxel-sensitive PCa cells; unpaired two-tailed student’s t-test. **(H)** Western blot and the quantitative results showing the expression level of HUWE1 protein between docetaxel-resistant and docetaxel-sensitive PCa cells; unpaired two-tailed student’s t-test. **P < 0.01, ***P < 0.001.

The original version of this article has been updated.

