# Supplementary figures and images for: PIGL promotes the docetaxel resistance of prostate cancer and is regulated by E3 ubiquitin ligase HUWE1
Source: Front Immunol. 2026 Jun 18;17:1740132. doi: 10.3389/fimmu.2026.1740132 (PMC13323019; doi:10.3389/fimmu.2026.1740132)

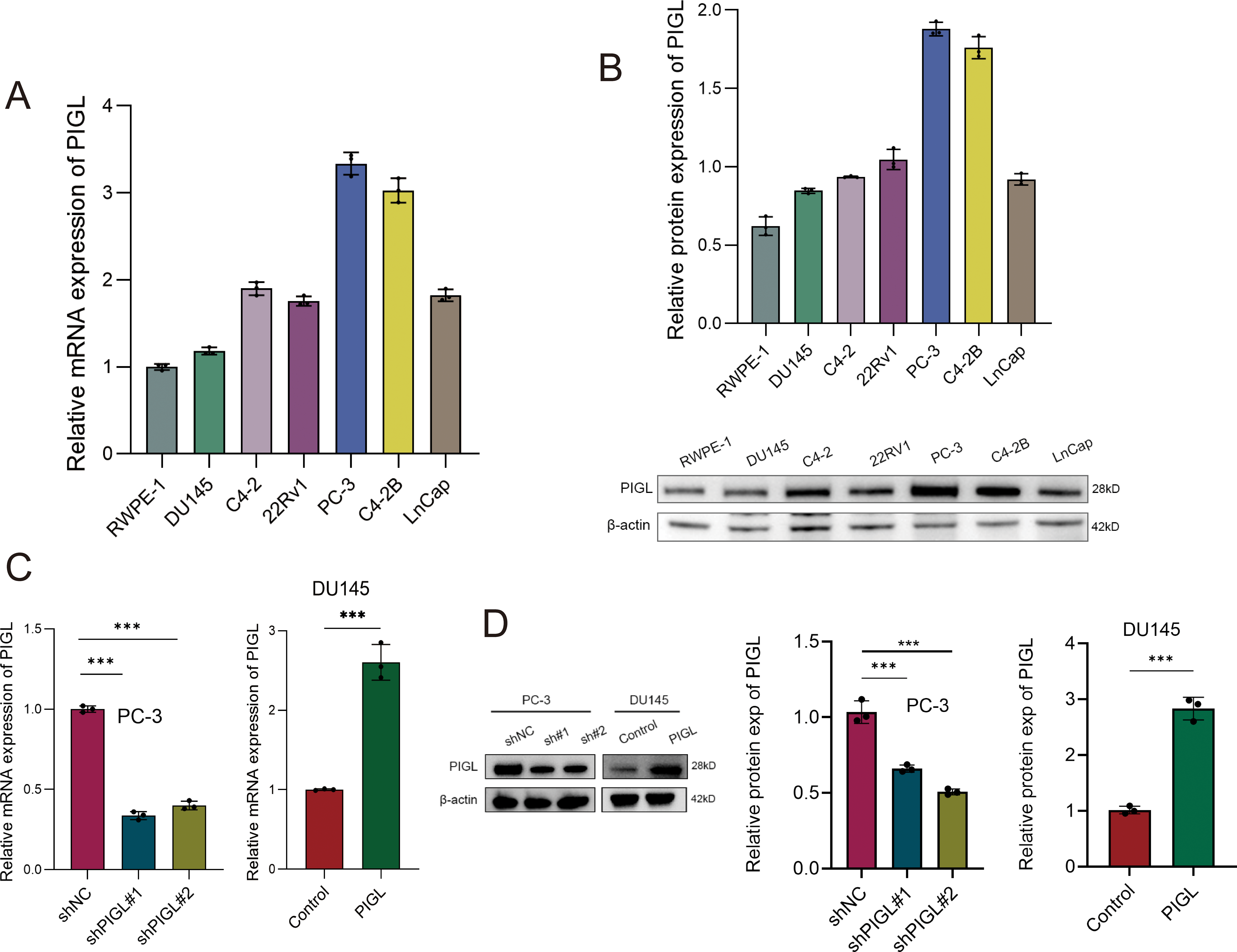

Supplement: Supplementary Figure 1 — The baseline expression and the efficiency of PIGL overexpression or knockdown in PCa cell lines. (A) RT-qPCR showing the expression level of PIGL mRNA in different PCa cells. (B) Western blot and the quantitative results showing the expression level of PIGL protein in different PCa cells. (C) RT-qPCR indicating the mRNA efficiency of PIGL overexpression in DU145 cells and PIGL knockdown in PC-3 cells; one-way ANOVA test and unpaired two-tailed student’s t-test. (D) Western blot indicating the protein efficiency of PIGL overexpression in DU145 cells and PIGL knockdown in PC-3 cells; one-way ANOVA test and unpaired two-tailed student’s t-test. *P < 0.05, **P < 0.01, ***P < 0.001. [file Image1.tif]

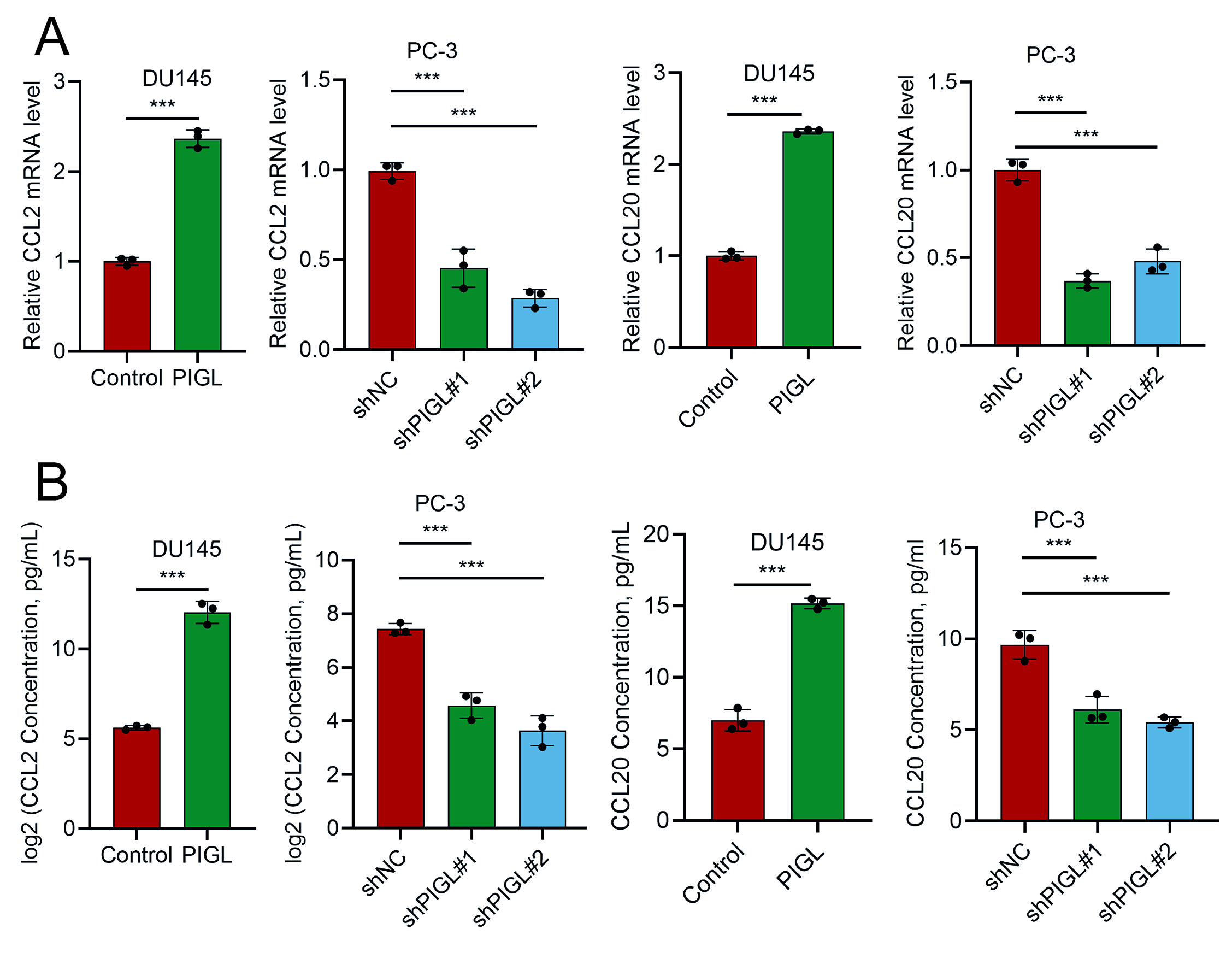

Supplement: Supplementary Figure 2 — The effect of PIGL on the expression of CCL2 and CCL20 in PCa cells. (A) The effect of PIGL on the expression of CCL2 and CCL20 mRNA in PCa cells.; unpaired two-tailed student’s t-test or one-way ANOVA test. (B) The effect of PIGL on the expression of CCL2 and CCL20 content in the culture medium of PCa cells.; unpaired two-tailed student’s t-test or one-way ANOVA test. [file Image2.tif]

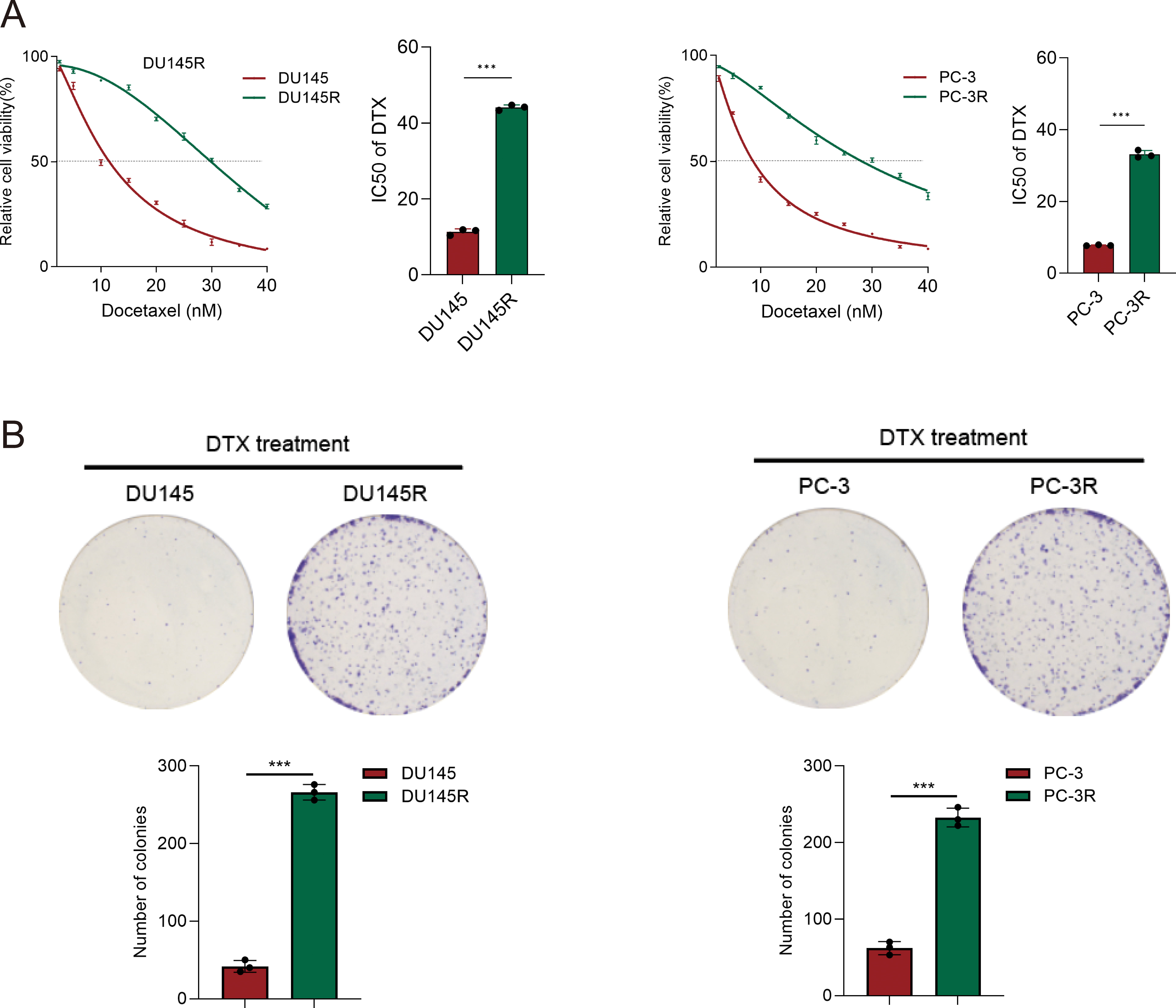

Supplement: Supplementary Figure 3 — Validation of the successful construction of docetaxel-resistant prostate cancer cells. (A) CCK8 toxicity assays showing the difference of the docetaxel IC50 between docetaxel-resistant and docetaxel-sensitive prostate cancer cells (PC-3R vs. PC-3; DU145R vs, DU145); unpaired two-tailed student’s t-test. (B) Representative images and quantitative results of cloning formation assay showing the difference of the clone formation capability between docetaxel-resistant and docetaxel-sensitive prostate cancer cells (PC-3R vs. PC-3; DU145R vs, DU145); unpaired two-tailed student’s t-test. *P < 0.05, **P < 0.01, ***P < 0.001. [file Image3.tif]

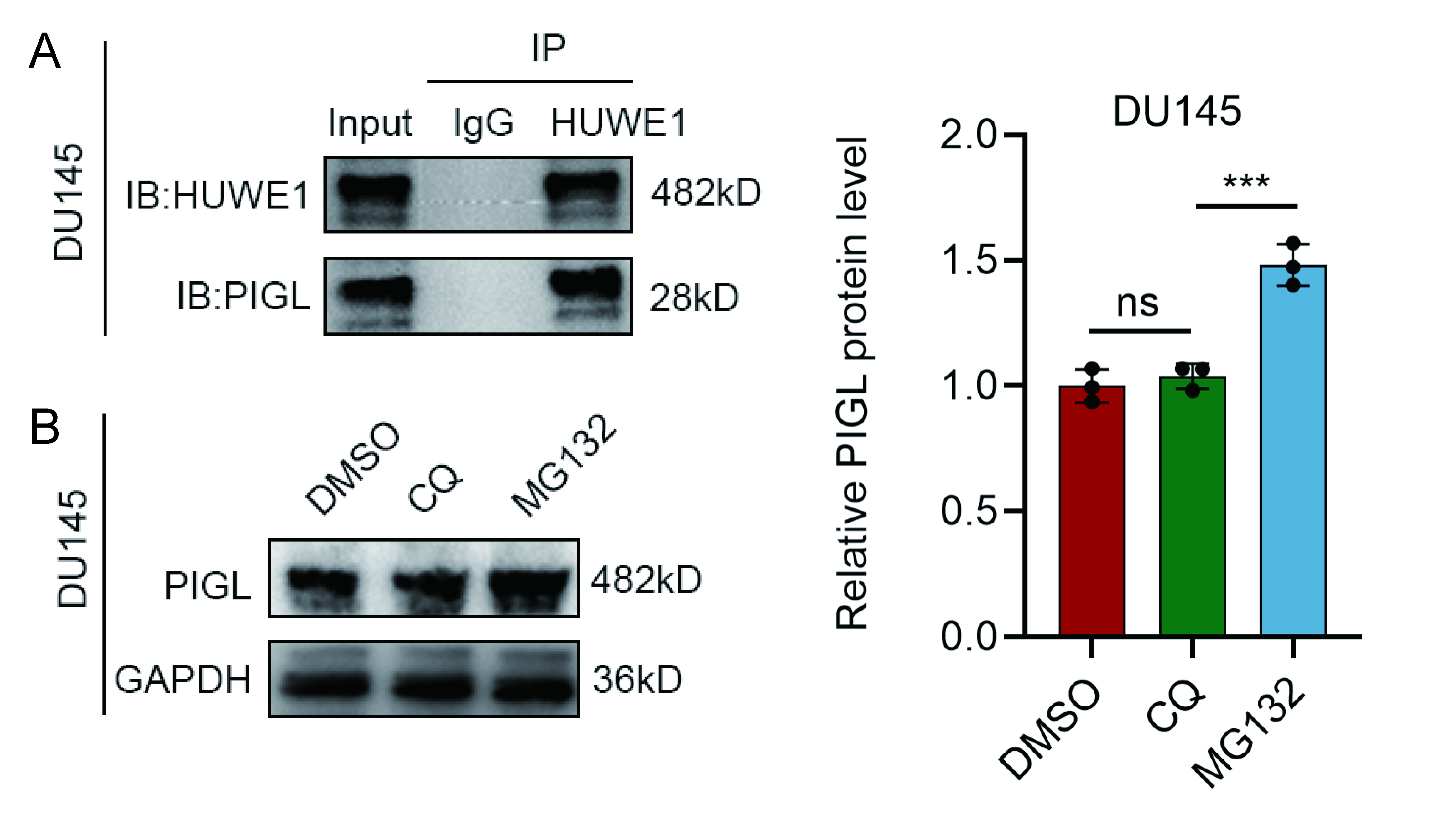

Supplement: Supplementary Figure 4 — The combination of PIGL and HUWE1 and the degradation pathway of PIGL protein. (A) co-immunoprecipitation assay followed by western blot showing the combination of PIGL protein and HUWE1 protein. (B) Western blot showing the expression levels of PIGL protein after treating with CQ or MG132; one-way ANOVA test. *P < 0.05, **P < 0.01, ***P < 0.001. [file Image4.tif]

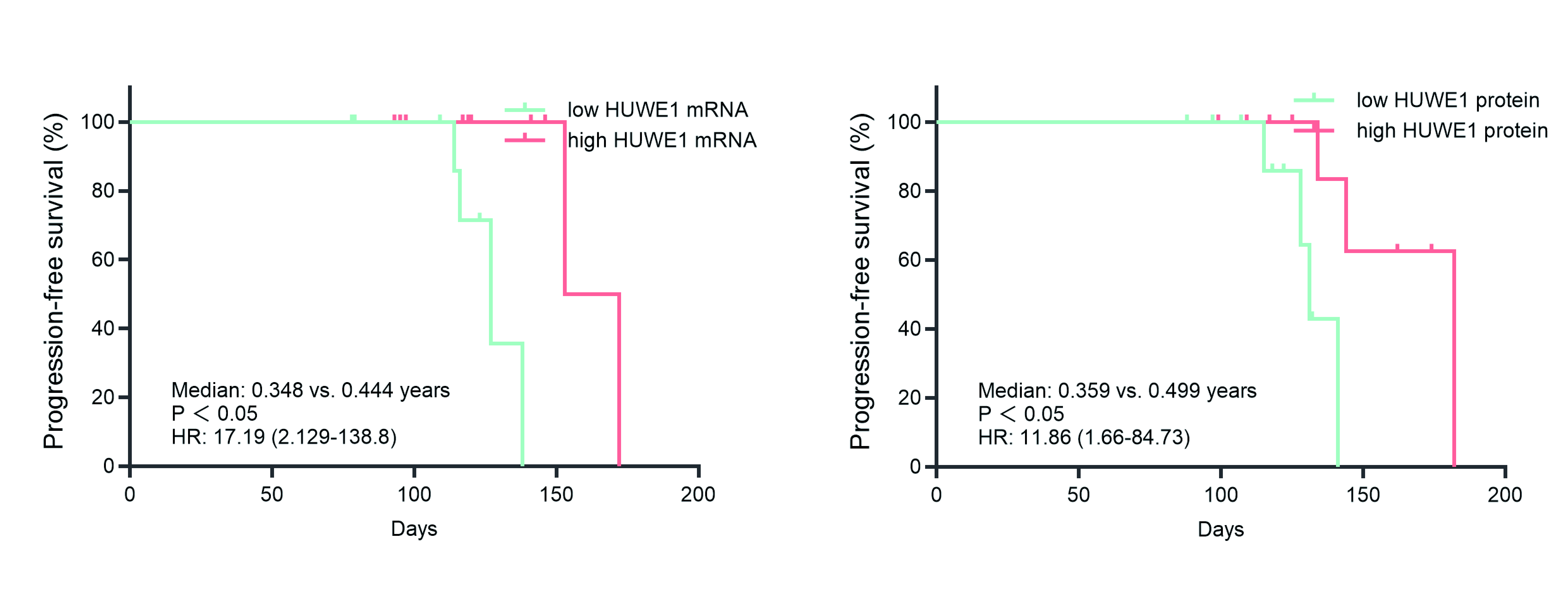

Supplement: Supplementary Figure 5 — The association of expression level of HUWE1 mRNA and protein with PFS in PCa biopsy tissues from mCRPC patients receiving docetaxel chemotherapy from another independent validation cohort of Second People’s Hospital of Yichang. [file Image5.tif]

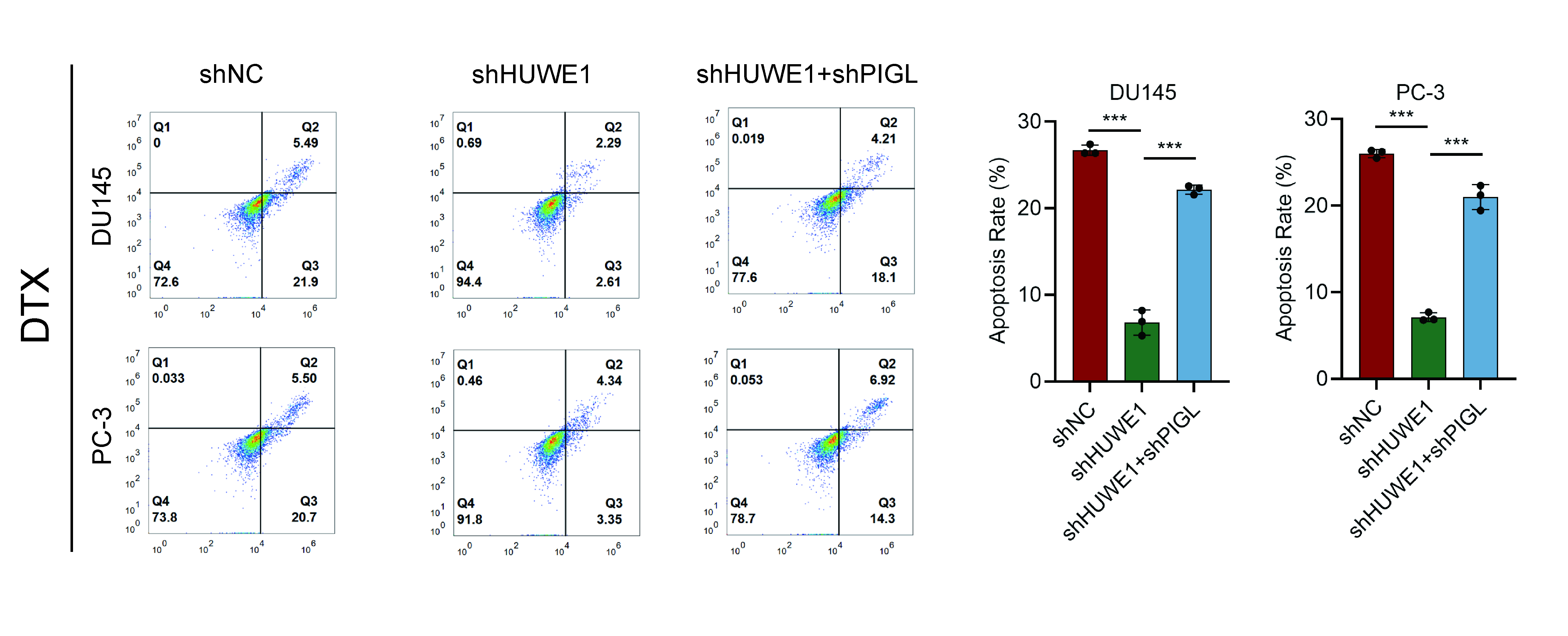

Supplement: Supplementary Figure 6 — Low expression of HUWE1 inhibits the apoptosis of PCa cells under docetaxel treatment. Flow cytometry apoptosis assay showed that the effect of HUWE1 knockdown on the apoptosis under docetaxel treatment of DU145 and PC-3 cells could be rescued by PIGL silencing; one-way ANOVA test. *P < 0.05, **P < 0.01, ***P < 0.001. [file Image6.tif]
